# Characterization and diversity of phages infecting *Aeromonas salmonicida* subsp. *salmonicida*

**DOI:** 10.1038/s41598-017-07401-7

**Published:** 2017-08-01

**Authors:** Antony T. Vincent, Valérie E. Paquet, Alex Bernatchez, Denise M. Tremblay, Sylvain Moineau, Steve J. Charette

**Affiliations:** 1 Institut de Biologie Intégrative et des Systèmes (IBIS), Université Laval, Quebec City, QC G1V 0A6 Canada; 2 Département de biochimie, de microbiologie et de bio-informatique, Faculté des sciences et de génie, Université Laval, Quebec City, QC G1V 0A6 Canada; 3 Centre de recherche de l’Institut universitaire de cardiologie et de pneumologie de Québec (IUCPQ), Quebec City, QC G1V 4G5 Canada; 4 Groupe de Recherche en Écologie Buccale (GREB), Faculté de médecine dentaire, Université Laval, Quebec City, QC G1V 0A6 Canada; 5 Félix d’Hérelle Reference Center for Bacterial Viruses, Faculté de médecine dentaire, Université Laval, Quebec City, QC G1V 0A6 Canada

## Abstract

Phages infecting *Aeromonas salmonicida* subsp. *salmonicida*, the causative agent of the fish disease furunculosis, have been isolated for decades but very few of them have been characterized. Here, the host range of 12 virulent phages, including three isolated in the present study, was evaluated against a panel of 65 *A. salmonicida* isolates, including representatives of the psychrophilic subspecies *salmonicida*, *smithia*, *masoucida*, and the mesophilic subspecies *pectinolytica*. This bacterial set also included three isolates from India suspected of being members of a new subspecies. Our results allowed to elucidate a lytic dichotomy based on the lifestyle of *A. salmonicida* (mesophilic or psychrophilic) and more generally, on phage types (lysotypes) for the subspecies *salmonicida*. The genomic analyses of the 12 phages from this study with those available in GenBank led us to propose an *A. salmonicida* phage pan-virome. Our comparative genomic analyses also suggest that some phage genes were under positive selection and *A. salmonicida* phage genomes having a discrepancy in GC% compared to the host genome encode tRNA genes to likely overpass the bias in codon usage. Finally, we propose a new classification scheme for *A. salmonicida* phages.

## Introduction

The Gram-negative bacterium *Aeromonas salmonicida* subsp. *salmonicida* is a major fish pathogen that is responsible for significant economic losses in the aquaculture industry worldwide^1^. More recently, genomic studies on *A. salmonicida* have led to a better understanding of its evolutionary history and global diversity, including its large number of mobile genetic elements^2–4^ as well as its lifestyle (psychrophilic or mesophilic)^4, 5^.

As with other bacteria, *A. salmonicida* subsp. *salmonicida* can be infected by viruses, namely bacteriophages or phages. Moreover, it is generally recognized that phages play significant roles in microbial ecology through bacterial lysis, reprograming of host metabolism and horizontal gene transfer^6–8^. One of the first studies on phages infecting *A. salmonicida* was published in 1933 and reported the presence of these biological entities in English rivers^9^. Another study on *A. salmonicida* phages published in 1970^10^ identified various lysotypes (i.e., groups of bacterial strains based on their sensitivity to specific phages), suggesting that *A. salmonicida* is subject to infection by dynamic and diverse groups of phages. It was later shown that the A-layer, a bacterial surface structure implicated in virulence^11^, may also be a phage receptor in *A. salmonicida* ^12^. Both virulent phages, which produce virions after lysis of the host cell, and temperate phages, which can integrate their genome into the host bacterial chromosome, have been isolated for *A. salmonicida* ^10, 13–19^.

With the rise of bacterial strains resistant to antibiotics, phages are now being revisited as a potential complement or alternative to antibiotics to treat bacterial infections^20^. For example, phages were explored to treat or prevent furunculosis, a disease caused by *A. salmonicida* subsp. *salmonicida* ^21^. Although phage therapy remains a promising strategy, there are potential drawbacks associated with the use of phages as antibacterial agents. For example, lysogenic conversion, the process whereby the host capitalizes on the genes encoded by prophage to enhance its own fitness, has conferred new capabilities in a wide range of bacterial species. It has now been established that some genes related to drug resistance^22^, metabolism^23^ and virulence^24^ in several bacteria are a result of lysogenic conversion. Thus, before using phages as therapeutics, it is important to characterize them at the genomic and phenotypic levels.

Although viruses are the most abundant biological entities on earth and are implicated in various important ecological processes^25^, their genomic sequences represent only a small fraction in public databases. For example, only 10 complete genome sequences of phages infecting *A. salmonicida* are currently available (phages 25, 56, 31, 65, 44RR2.8t, Aes508, AS4, AS5, AS7, and PX29). These phages are classified in the *Myoviridae* family (dsDNA genome, long contractile tail), except phage AS7, which is a member of the *Podoviridae* family (dsDNA genome, short tail). The genomes of five *Aeromonas* phages (Aeh1, Aes012, CC2, pAh6-C, ΦO18P) infecting other species (*hydrophila, media*) are also available in GenBank.

Based on comparative genomic analyses with other phages^17, 26^, *A. salmonicida* phages have been classified by the International Committee on Taxonomy of Viruses (ICTV) into several different taxa. For example, the *Secunda5virus* genus of the *Myoviridae* family includes the viral species *Aeromonas virus 25, Aeromonas virus 31, Aeromonas virus Aes508*, and *Aeromonas virus AS4*. The *Biquartavirus* genus of the *Myoviridae* family comprises the species *Aeromonas virus 44RR2.8t*. In addition, there are several species of *Aeromonas* myophages that have yet to be assigned a genus, such as *Aeromonas virus 65*.

Here, we have significantly increased the genomic information of *Aeromonas* phages. The complete genomes of 12 phages infecting *A. salmonicida* subsp. *salmonicida* were obtained and analyzed. The antimicrobial potential of these phages was also evaluated against a panel of 65 strains of *A. salmonicida*. Additionally, we propose a revised classification scheme for phages infecting *A. salmonicida* subsp. *salmonicida*.

## Results and Discussion

### *A. salmonicida* phages

**Figure 1:**
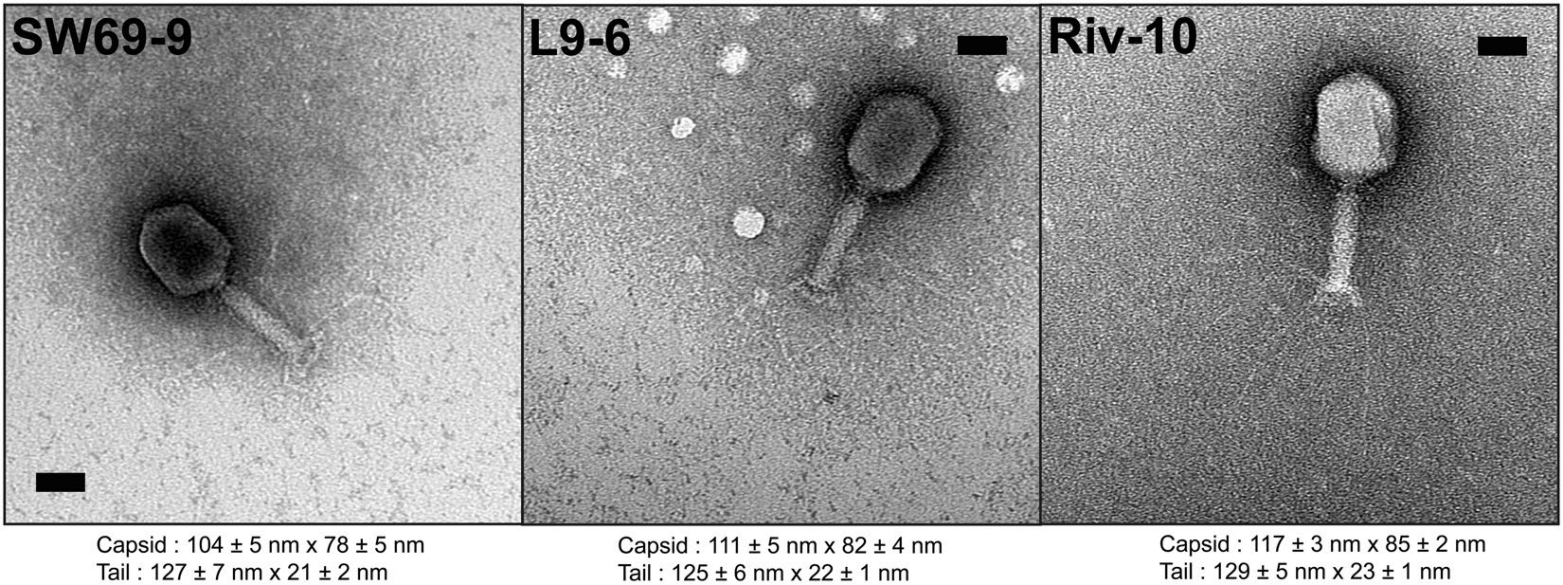
Micrographs of phages SW69-9, L9-6 and Riv-10. The average head/tail length and diameter are indicated below each phage. The bars represent 50 nm.

One of the goals of this study was to isolate and characterize new virulent phages able to infect *A. salmonicida* subsp. *salmonicida* and to assess the diversity of phages infecting this species. Water samples were collected from three rivers, including two passing through fish farms within the Province of Quebec (Canada). All samples tested were found to contain *A. salmonicida* subsp. *salmonicida* phages. Three phages were purified and named SW69-9, L9-6 and Riv-10. As shown in Fig. 1, the three phages belong to the *Myoviridae* family with an elongated capsid and a contractile tail. As determined by one-step growth curves (see Supplementary Fig. S1), the burst sizes were very low at 3, 2 and 2 new virions per infected cell for phages SW69-9, L9-6 and Riv-10, respectively while the latent period was long at 145, 150 and 142 minutes. Such low burst sizes were not unexpected since *Aeromonas virus 31*, which is genetically close (see Pan-genome analysis section), is known to have a burst size of 7 new virions per infected cell^27, 28^.

Nine other virulent phages infecting *A. salmonicida* were obtained from the Félix d’Hérelle Reference Center for Bacterial Viruses (phage 3 (HER84), 31 (HER105), 32 (HER106), 51 (HER108), 56 (HER109), 59.1 (HER100), 65 (HER110), 44RR2.8t (HER98), and Asp37 (HER99)). The electron micrographs of these phages are available online on the Félix d’Hérelle Reference Center website (http://www.phage.ulaval.ca) and are all classified in the *Myoviridae* family.

### Genomic characterization

The complete genomes of the 12 phages (9 from the Félix d’Hérelle Reference Center for Bacterial Viruses and the 3 phages isolated from the environmental samples in this study) were sequenced using Illumina technology and *de novo* assembled, resulting in a final database of 18 *A. salmonicida* subsp. *salmonicida* phage genomes, which includes the six genomes already available through GenBank, namely the podophage AS7 and the myophages 25, Aes508, AS4, AS5, and PX29 (Table 1). It should be noted that we resequenced the genomes of phages 31, 56 [deposited as vB_AsaM-56], 65, and 44RR2.8t and subsequently renamed them 31.2, 56, 65.2 and 44RR2.8t.2. Non-synonymous mutations were present between the two versions of the four genomes (see Supplementary Table S1), suggesting that either these phages have evolved or that these discrepancies are due to the use of a different sequencing technology and genome assembly software.

**Table 1:** Phages infecting *A. salmonicida* used in this study.

Name	HER^a^	Taxonomy	Isolation	Genome size (bp)	CDSs	tRNAs	GC%	Integrase	GenBank	Reference
**Phages having a small genome**										
AS7	N/A^b^	*T7-like* ^c^	N/A	41,572	53	0	57	No	JN651747.1	13
51	108	*Myoviridae*	France	43,551	84	0	55	Yes^d^	KY290953	10
56	109	*Myoviridae*	France	43,551	84	0	55	Yes^d^	KY290954	10
59.1	100	*Myoviridae*	Canada	46,057	87	0	54	Yes	KY290950	14
3	84	*Myoviridae*	France	46,349	83	0	57	Yes	KY290947	10
Asp37	99	*Myoviridae*	Canada	47,977	83	0	57	Yes	KY290949	14
32	106	*Myoviridae*	France	48,252	83	0	57	Yes	KY290952	10
**Phages having a medium genome**										
Aes508	N/A	*Myoviridae* (*Secunda5virus*)	N/A	160,646	230	10	41	No	JN377894.1	N/A
25	85	*Myoviridae* (*Secunda5virus*)	France	161,475	232	11	41	No	DQ529280.1	10
AS4	N/A	*Myoviridae* (*Secunda5virus*)	Korea	163,875	268	15	41	No	HM452125.1	15
44RR2.8t.2	98	*Myoviridae* (*Biquartavirus*)	Canada	173,590	253	16	44	No	KY290948	14
31.2	105	*Myoviridae* (*Secunda5virus*)	France	172,957	245	16	44	No	KY290951	10
SW69-9	523	*Myoviridae*	Canada	173,097	249	16	44	No	KY290958	This study
L9-6	524	*Myoviridae*	Canada	173,578	251	16	44	No	KY290956	This study
Riv-10	525	*Myoviridae*	Canada	174,311	249	16	44	No	KY290957	This study
**Phages having a large genome**										
PX29	N/A	*Myoviridae*	N/A	222,006	322	24	42	No	GU396103.1	17
AS5	N/A	*Myoviridae*	Korea	225,268	333	25	43	No	HM452126.1	16
65.2	110	*Myoviridae*	France	236,567	410	18	37	No	KY290955	10

^a^Refers to the Felix d’Hérelle collection (http://www.phage.ulaval.ca) number. ^b^Means none applicable. ^c^Only based on bioinformatics inference. ^d^Low confidence in the gene’s identity.

A striking feature was the pronounced divergence in GC% between phages with small (56% ± 1.29) or medium/large (42.27% ± 2.19) genome (Table 1). These results were surprising because in general the GC% of phage genomes tend to correspond with the GC content of their bacterial hosts^29^, which in the case of *A. salmonicida* subsp. *salmonicida*, is 58.5%. A previous study on codon co-evolution showed that *A. salmonicida* phage genomes with small GC% encode tRNA genes that permit the phages to presumably bypass codons overused in the phage genes, since the repertoire of tRNAs from the host is likely inadequate to translate efficiently phage mRNAs^30^. Our larger dataset of phages allowed us to investigate whether phages with low or high GC% can be statistically differentiated based on (1) the relative synonymous codon usage (RSCU) and (2) the amino acids composition. Principal component analysis (PCA) showed in both cases a clear separation between genomes having low or high GC% (see Supplementary Fig. S2). Interestingly, although the genome with the lowest GC% (phage 65.2 with 37%) generally clustered with other low-GC% genomes (see Pan-genome analysis section), it was a distinct outlier, which is concordant with its extreme GC%. Also, an analysis of our dataset showed a perfect correlation between the presence or absence of tRNA genes and the GC% of the genomes (Table 1).

### Pan-genome analysis

The pan-genome (i.e., whole gene repertoire of a study group) of our current phage dataset was determined. An analysis that included all the ORFs from all 18 phage genomes (3,599 sequences) grouped them into 1,222 clusters. As expected considering phage diversity, no cluster contained ORFs from all phage genomes. Consequently, there is no core-genome when one takes all 18 genomes into consideration (see Supplementary Fig. S3). Although via manual curation we were able to identify a large terminase subunit that was present in all of the genomes, the percent sequence similarity was so low between some sequences (see Supplementary Fig. S4) that they were not considered as homologous by GET_HOMOLOGUES (see Methods section). However, even with a high degree of sequence divergence, phylogenetic clusters based on large terminase subunit sequences are well known to correlate with DNA packaging strategies^31^. We assembled a database of the large terminase subunit sequences of the 18 *A. salmonicida* phages from this study and 78 other terminase sequences from phages known to have different DNA packaging strategies, many of which were experimentally validated, to generate a large-scale molecular phylogeny (see Supplementary Fig. S5). The results of this analysis suggest that most of the 18 phages from our study have a headful packaging strategy, although one phage (AS7) has short direct terminal repeats (DTRs) and four (3, Asp37, 32 and 59.1) use 5′ protuberant *cos* ends. These findings support the view that a diverse phage population infects strains of *A. salmonicida*.

**Figure 2:**
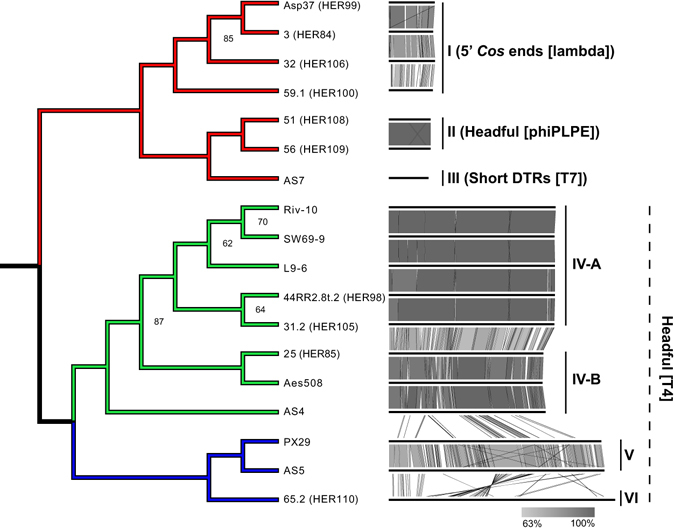
Clustering based on the gene repertoire. Phages having a small, medium and large size genome are in red, green and blue, respectively. The AU (Approximately Unbiased) *p*-value is indicated at each node when inferior to 100. Alignment of genomes and the proposed grouping are coupled to the tree. The inferred mode of DNA packaging is indicated for each cluster.

However, it also complicates their phylogenetic analysis because the sequences do not share a sufficient number of valuable sites to infer robust relative phylogenetic positions among the phages without introducing a clear functional bias. Even by using a Bayesian phylogeny approach with the site-heterogeneous model CAT^32^, an approach known to reduce artefacts due to long branch attraction^33^, we recovered a tree topology with poor statistical support where the podophage AS7 was basal to the clade comprised of phages with medium and large genomes (see Supplementary Fig. S6A). We assessed the potential saturation of the phylogenetic matrix by plotting the uncorrected p-distance with the pairwise distances in branch lengths from the tree^34^. Since the resulting plot clearly showed a non-linearity, we concluded that any true evolutionary signals were obfuscated by saturation and homoplasy (see Supplementary Fig. S6B). In order to bypass this issue, we encoded the pan-genome matrix generated by the previous analysis in binary (i.e. presence/absence of gene clusters) and then performed hierarchical clustering (Fig. 2). The nodes within this tree were well supported (Approximately Unbiased (AU) p-value greater than 95), with some exceptions. With this approach, the phage genomes clustered based on their size (small: 45,329 bp ± 2,502; medium: 169,191 bp ± 6,036; large: 227,947 bp ± 7,641). By coupling this tree with a sequence alignment (Fig. 2), we were able to classify the phages into six groups (I to VI), with the fourth group further divided into subgroups A and B. We supported this grouping by combining the tree with a resulting identity matrix based on BLASTP (see Supplementary Fig. S7). Interestingly, phages also clustered together based on their predicted mode of DNA packaging (Fig. 2). These results show that even if phages infect the same host, they may exhibit considerable genomic diversity.

The group III contained the only *Aeromonas* phage of the *Podoviridae* family. Our phylogeny clustered, *Aeromonas viruses 25, Aes508*, and *AS4* into the same subgroup (IV-B), indicating that they are closely related, and likely in the same species. In contrast the ICTV has classified these three phages as different species albeit in the same genus (*Secunda5virus*). Moreover, *Aeromonas viruses 31.2* and *44RR2.8t.2*, which are also considered different species by the ICTV, clustered in subgroup IV-A along with the three new phages isolated in this study (Riv-10, SW69-9, and L9-6). As shown in Fig. 2 and with a focus on the group IV-A (see Supplementary Fig. S8), the new isolated phages share genomic features, even if they were isolated from various sites within the Province of Quebec. It is also interesting to note that the three new phages from the Province of Quebec are as genetically distant between them (~97.8% of whole genome identity) as *Aeromonas virus 31.2* is from *44RR2.8t.2* (97.2% of whole genome identity). The latter were isolated in France and Ontario, respectively (see Supplementary Fig. S9) and are the most similar phage genome sequences available in GenBank. Phages Asp37, 3, 32 and 59.1 were clustered in group I, while phages 51 and 56 were combined in group II. Phages PX29 and AS5 were found in group V and finally, phage 65.2 was placed in group VI. Clearly, ICTV uses a very stringent approach to speciate phages, which otherwise seem highly similar at the genomic level. A 95% DNA sequence identity (BLASTN algorithm) was apparently chosen by the ICTV as the criterion for demarcation of species in the *Secunda5virus* genus.

Because no gene clusters were shared by all the genomes, we identified the core-genome for each group and verified the number of shared core-clusters among them. We were unable to identify a core cluster among the small genome phages (I, II and III) or shared between the small and the medium size phage genomes (IV-A and IV-B), underlining the extreme sequence heterogeneity among these viruses (see Supplementary Fig. S10). Additionally, we could not identify a core gene cluster among the small genomes and group V, a group that contains phages with larger genomes. There is a small core-cluster, however, shared between the genome of phages AS7 (III) and 65.2 (VI). This is surprising given that AS7 belongs to the *Podoviridae* family while 65.2 is a myophage. The shared gene encodes a hypothetical protein without any known function. A BLASTP analysis uncovered an orthologous gene in the genome of CC2, a phage that infects *Aeromonas hydrophila* ^35^. But again, this putative phage protein has yet to be assigned a function.

**Figure 3:**
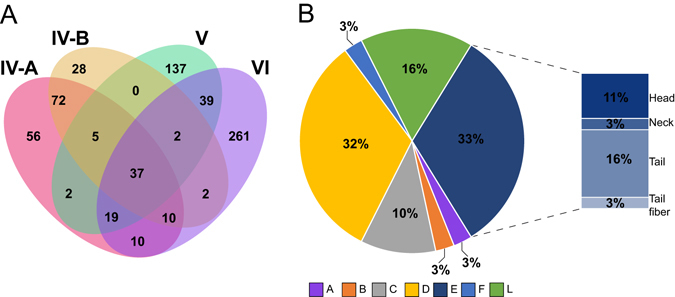
Shared core-clusters analysis. The shared core-clusters between medium (IV-A and IV-B) and large (V and VI) genomes are represented as a Venn diagram (**A**). The 37 core-clusters shared by the medium- and large-size genomes were grouped by functional categories (**B**). The categories are: (A) Transcription, (B) Translation, (C) Nucleotide metabolism, (D) DNA replication, recombination, repair, packaging, and processing, (E) Virion proteins, (F) Chaperonins/assembly catalysts and (L) Others.

The medium and large size genomes are less heterogeneous in their gene repertoire and thus allowed us to identify a larger core genome (Fig. 3A). Our analysis identified 37 clusters that were shared amongst medium and large genomes. These genes were grouped by functional categories accordingly to another study (Fig. 3B)^36^. More than half (~66%) of the coding sequences (CDSs) can be grouped into two categories: non-structural (i.e. DNA replication, recombination, repair, packaging), and structural (capsid, tail etc.) proteins. Interestingly, 16% of the CDSs could not be assigned to a functional category. These CDSs included four hypothetical proteins, a putative nicotinamide phosphoribosyl transferase and a lysozyme.

### Genes under positive selection

Our next objective was to investigate whether the orthologous genes that we had identified in the previous analysis were under positive selection (also known as diversifying or Darwinian selection). A standard procedure to quantify the selection of a gene is by calculating the d*N*/d*S* ratio, d*S* being the synonymous substitution rate (assumed to be neutral) and d*N* being the non-synonymous substitution rate (an indicator of positive selection since the amino acid composition of the encoded protein was modified)^37^. When two phages co-infect a bacterial cell, their genomes may exchange genetic segments through recombination^38^. Recombination can drive genome evolution but also bias in the detection of selection by increasing the number of false positively selected sites^38^. We evaluated the recombination events and partitioned each gene based on where these events may have taken place. We then used an algorithm optimized to infer positive selection from the recombining coding sequences identified in the previous analysis.

Our results identified five phage genes that were significantly (*p* < 0.05) under positive selection (Table 2). The gene under positive selection with the smallest *p*-value was *ndd*, a gene that encodes a protein implicated in the disruption of the bacterial nucleoid^39^. The second most significant gene under positive selection was gene *6*, encoding the baseplate protein gp6. This protein is likely present in multiple copies and forms a continuous ring around the central hub while playing a critical role in the assembly and function of the baseplate^40, 41^. During the infection of an *Escherichia coli* cell by the myophage T4, six long-tail fibers interact reversibly to the cell-surface of the host, followed by the attachment of short-tail fibers that bind to additional receptors on the cell surface in an irreversible fashion. The attachment of the short-tail fibers triggers a conformation change of the baseplate from a dome-shaped to a star-shaped^42^. Recent studies have shown that gp6 is one of the key proteins in the signal transmission from the short-tail fibers to the central region of the baseplate during this conformational shift^40, 41^. Gene *6* was found in medium and large size genomes, with the exception of phage AS4. Deeper investigation led to the identification of a *6-like* gene within the genome of AS4, but with multiple frameshifts. It is tempting to speculate that these frameshifts may be the result of sequencing artefacts given the vital role of gp6. It is not clear why gene *6* is under positive selection, but if gp6 is implicated in host infection as it appears, mutations to this gene may alter attachment kinetics of the phage to the cell surface, leading to changes in host specificity.

**Table 2:** Genes under positive selection.

Gene name	Protein	*p* value	Clusters
*ndd*	Host nucleoid disruption protein	0.0096557	IV-A, IV-B
*6*	gp6 base plate wedge component	0.0126069	IV-A, IV-B^a^, V, VI
N/A	Hypothetical protein^b^	0.0143135	I
*44*	gp44 clamp-loader subunit	0.0212075	IV-A, IV-B, V, VI
*60plus39*	Topoisomerase II large subunit	0.04291	IV-A, IV-B, V, VI

^a^The gene contains multiple frameshifts for AS4 and was consequently not added to the analysis. ^b^ *de novo* predicted by the present study.

The last three genes under positive selection code for a hypothetical protein, the clamp-loader subunit gp44 and a topoisomerase II large subunit. It is worth noting that the gene encoding the hypothetical protein is the only gene from phages with small genomes found to be under positive selection. Additionally, the topoisomerase II large subunit is the product of two genes (*39* and *60*) in phage T4^43^ and thus may be a genomic region under disproportionate positive selection.

### Host range of the phages

Temperate phages are able to integrate their genomes into the chromosome of their host. In some instances, these phage-encoded genes are used by the host to its own advantage. We investigated each of the 18 genomes for the presence of an integrase gene, the hallmark of temperate phages. A gene coding for an integrase was found in all the genomes of phages in group I (3, Asp37, 59.1, and 32). This suggests that these phages are likely capable of lysogeny and are therefore not good candidates for phage therapy. The genomes of phages 51 and 56, both from the group II, appeared to harbour a gene encoding for a truncated integrase, however we have low confidence in the gene’s identity. The podophage AS7 (group III) is the only phage having a small genome that does not harbour a gene encoding for an integrase. No genes coding for antibiotic resistance and/or virulence factors were identified in any genome.

**Figure 4:**
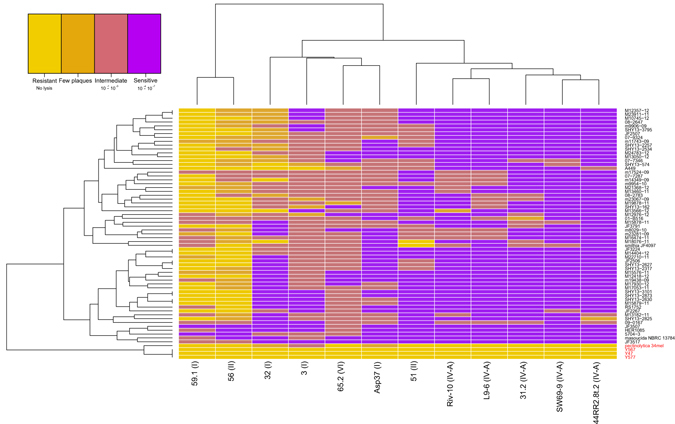
Clustering and heatmap based on a panel of 65 *A. salmonicida* isolates challenged with 12 phages. The genomic cluster of each phage is indicated in parentheses. The mesophilic isolates are shown in red while the psychrophilic ones are in black. High and low lytic activities are represented in purple and yellow, respectively. In addition, the dilution ranges obtained by spot tests used to encode the matrix are indicated below the legend.

We assessed the host range of the 12 phages with a panel of 65 *A. salmonicida* isolates (Fig. 4). To investigate the specificity of these phages, isolates from subspecies other than *salmonicida* were added: *smithia*, *masoucida*, *pectinolytica* as well as three Indian strains suspected of being part of a new *A. salmonicida* subspecies^4^. The resulting host range patterns coupled to a clustering (heatmap) allowed us to classify the phages into three groups.

The first group contained phages (phages 59.1 (genomic cluster I) and 56 (II)) with the narrowest host range, where only few bacterial isolates (<5 out of 65) were sensitive to the phages based on spot tests (Fig. 4). As indicated previously, the genome of phage 59.1 possesses an integrase gene while it is not clear if phage 56 has a functional integrase gene. Neither of these phages was reported as temperate by the original studies that characterized them^10,14^. However, the bioinformatics tool PHACTS^45^, which predict the lifestyle of a phage, indicated that these two phages are likely temperate.

The second group, composed of phages 32 (group I), 3 (group I), 65.2 (group VI) and Asp37 (group I), displayed an intermediate host range where 10 to 30 bacterial isolates were sensitive to the phages (Fig. 4). Phages 32, 3 and Asp37 have a gene encoding for an integrase, consequently with the potential to be temperate under some conditions. Even though we were unable to identify a gene coding for an integrase in the phage 65.2 genome, it has the highest GC% discrepancy (37%) with the genome GC% of its host (58.5%), raising the possibility that this phage is less efficient at lysing its host.

The third group included phages within the genomic cluster group IV-A and phage 51 (cluster II). Members of this group had the broadest host range (>44 out of 65) (Fig. 4). Consequently, phages from this group have the most potential from a phage therapy perspective. For example, phage 44RR2.8t.2 infected 57 out of the 65*A. salmonicida* strains. Similarly, the newly isolated phage SW69-9 infected 56 out of the 65 strains. The host range of phages 44RR2.8t.2 and SW69-9 was overlapping and as such, all *A. salmonicida* subsp. *salmonicida* strains could be infected by one of the two phages or both (Fig. 4).

Surprisingly, phage 51 was able to lyse 41 strains, even if the original study reported it to be temperate^10^. The tool PHACTS also predicted the lifestyle of this phage as temperate, but with an uncertain probability (0.515 ± 0.042). This was even more surprising because the other phage within the cluster II, phage 56, had a very limited host range (lysed 4 strains). A global alignment of both phage genomes (51 and 56) resulted in only four non-synonymous SNPs. The first two were located in genes coding for hypothetical proteins, each having a predicted conserved unknown domain (DUF2213 and DUF2184). While the last two SNPs are in a gene encoding for a tail or truncated integrase protein. One SNP led to change from a methionine to a valine and the other one from a proline to leucine. Further work is required to determine which mutation(s) is responsible for the expanded host range.

Strains belonging to other psychrophilic subspecies (*smithia* and *masoucida*) were also sensitive to phages infecting the subspecies *salmonicida*. However, the three mesophilic strains from India^4^ (Y577, Y567 and Y47) and the strain of the subspecies *pectinolytica* (also having a mesophilic lifestyle^5^) were insensitive to all 12 phages, at the exception of *pectinolytica*, which is sensitive to phage 3. The A-layer, which is a structure composed of a protein and lipopolysaccharides implicated in virulence^11^, could be one of the phage receptors in *A. salmonicida* ^12^. Analysis of the various bacterial genomes showed that the gene *vapA*, encoding the protein forming the A-layer, is present in psychrophilic isolates but absent in mesophilic isolates. In order to better understand host phage dynamics in mesophilic *A. salmonicida*, it could be interesting to isolate phages able to infect them and to identify the phage receptor.

It is well documented that prophages can provide resistance to infection from other phages, by superinfection exclusion (Sie) systems found in both Gram negative and positive bacteria^46^. At the exception of the mesophilic/psychrophilic separation, the heatmap in Fig. 4 shows no clear bacterial clustering suggesting that such resistance could be provided (see Supplementary Fig. S11). This is of interest because *A. salmonicida* subsp. *salmonicida* isolates usually harbour two prophages sharing structural similarities with the temperate phage ΦO18P ^47^, found in *Aeromonas media*. Moreover some *A. salmonicida* subsp. *salmonicida* strains also have a new recently discovered prophage (prophage 3) and variants of a genomic island named *AsaGEI* ^2, 3, 48^. None of these mobile elements actually have a known function and we can reasonably here rule out their implication in protection against phages.

In conclusion, we investigated the genomes of 18 phages infecting *A. salmonicida* and characterized their diversity, which led to a robust classification scheme based on their genomic composition. We argue that our approach to classifying these phages will result in a more accurate characterization and classification of new *A. salmonicida* phages in the future. We also evaluated the infectivity of 12 phages, including three newly isolated phages, on a panel of 65 isolates of *A. salmonicida*. Overall, these phages showed a heterogeneous host range. Phages with overlapping and large host range were identified and hold potential to contribute to a phage cocktail to control this fish pathogen in the aquaculture industry.

## Methods

### Environmental sampling, phage isolation, and characterization

Skin mucus samples from furunculosis-infected fishes from four fish farms in the province of Quebec (Canada) as well as water samples (50 ml) from the same fish farms and rivers in the same region were collected and kept at 4 °C. The mucus was swabbed and diluted in 5 ml of phage buffer (50 mM Tris-HCl pH 7.5, 100 mM NaCl, 8 mM MgSO_4_). Each environmental sample was centrifuged for 10 min at 3,200 × *g* and filtered (0.45 μm, Sarstedt, Canada). The filtrate was then mixed with the same volume of 2X TSB (EMD Millipore, Canada) and inoculated with 1% (v/v) of *A. salmonicida* subsp*. salmonicida* in exponential growth phase. The bacterial strains used were those recommended by the Felix d’Hérelle Reference Center for Bacterial Viruses (http://www.phage.ulaval.ca). All cultures were incubated at 18 °C overnight and agitated at 200 RPM before being filtered again. The above amplification procedure was repeated four times or until the appearance of cell lysis (replacing 2X TSB with 1X TSB after each round). In parallel, controls without environmental sample were inoculated with the bacterial host to compare the growth and whether lysis had occurred in challenged host cultures.

The new phages were isolated from single plaques as follows: in 3 ml of TSB soft agar (0.75%) kept at 55 °C, 100 μl of filtrate and 100 μl of bacterial host (*A. salmonicida* subsp. *salmonicida* 08-2783 host of L9-6 and *A. salmonicida* subsp. *salmonicida* M15879-11 host of SW69-9 and Riv-10) were mixed and poured onto TSA plates (EMD Millipore, Canada) before being incubated at 18 °C overnight. Plaques (up to 10 per sample) were picked up with sterile tips and suspended in 500 μl of phage buffer. Phages were allowed to diffuse for 30 min at room temperature before serial dilutions in phage buffer. The above plaque isolation procedure was repeated two more times. Subsequently, phages were amplified in 10 ml of TSB incubated with bacterial host at 18 °C overnight and agitated at 200 RPM. Aliquots of the resulting filtrates were either stored at −80 °C in 15% glycerol or stored at 4 °C for up to six months until subsequent analysis.

Transmission electron microscopy was used to observe the three new phages as described elsewhere^49^. Briefly, 1.5 mL of phage lysate was centrifuged at 23,500 × *g* for 1 h at 4 °C and the pellet washed twice with ammonium acetate (0.1 M, pH 7.0). The resulting phage preparation was then used to prepare observation grids, which were stained with uranyl-acetate (2%) and observed with a JEOL 1230 at the microscopy platform of the Institut de Biologie Intégrative et des Systèmes (U. Laval). Capsid size and tail length were determined by measuring at least 15 different specimens.

Phage one-step growth curve assays of phages were performed in triplicate, as previously reported elsewhere^50^. Approximately 10^9^ CFU/ml of precultured cells that just reached stationary phase were harvested by centrifugation and resuspended in 900 µl of TSB. Each phage was respectively added at a multiplicity of infection (MOI) of 0.05 and allowed to adsorb for 20 minutes at 18 °C. Then, infected cells were harvested by centrifugation and the pellet washed twice with 1 ml of fresh TSB. Finally, the pellet containing the infected cells was resuspended in a final dilution of 0.001 in a glass tube containing 10 ml of TSB and incubated at 18 °C at 200 RPM. Every 30 min, an aliquot of 100 µl was taken to determine the phage titer, up to 210 minutes. The burst size was calculated by dividing the average phage titer after the exponential phase by the average titer before the infected cells began to release virions^51^. The latent period was evaluated according to the median of the exponential curve.

The DNA of the amplified phages was extracted using a standard phenol/chloroform protocol and then analyzed by restriction profiles using the enzymes DraI, SspI and MseI (NEB, Canada) according to the manufacturer recommendations at 37 °C for one hour. Restricted phage DNA was run on a 1% agarose gel for 30 min at 90 V. Three different restriction profiles were observed and the distinct phages were named SW69-9, L9-6 and Riv-10.

### Sequencing and *de novo* assembly

Sequencing libraries were prepared from purified phage DNA using the Nextera XT DNA Library Preparation Kit and sequenced on an Illumina MiSeq apparatus. The resulting sequencing reads were *de novo* assembled by the pipeline A5-miseq version 20150522^52^ to obtain an initial sequencing depth. Given their small size and the high-throughput capabilities of the Illumina platform, viral genomes are usually sequenced with very high depth, causing an unnecessary complexity for subsequent *de novo* assembly^53^. Consequently, the reads of each sequenced genome were randomly down-sampled by seqtk (https://github.com/lh3/seqtk) to obtain assemblies (also with A5-miseq version 20150522) having around 100X of sequencing depth.

We compared the resequenced phage genomes 44RR2.8t, 31, 56 and 65 by generating kmers with a length of 300 nt and then mapping them with BWA version 0.7.12-1039^54^ to reference sequences. Mutations were called by using samtools version 0.1.19-44428cd^55^ and VarScan version 2.4.2^56^. Finally, the effect of mutations was evaluated based on SnpEff version 4.2^57^ (with the reference sequences added beforehand in the database).

### Annotation, pan-genome and other bioinformatics analyses

Each genome, including those already available in GenBank, was annotated through the webserver RAST^58^ by choosing the “virus” parameter. A list of all predicted genes is available for each new phage: SW69-9, L9-6 and Riv-10 (see Supplementary Table S2). Annotated CDSs were downloaded and evaluated by GET_HOMOLOGUES version 2.0.16^44^. The sequences were clustered by using the COG and OMCL algorithms, both included in GET_HOMOLOGUES. Only the clusters found by both algorithms were used for downstream analyses (see Supplementary Fig. S12). The presence/absence binary matrix was evaluated by Pvclust^59^ under the binary distance method with 10,000 bootstrap replicates through the statistical framework R (https://www.r-project.org) resulting in a clustering of the phages based on their gene repertoire. An identity matrix was calculated with the BLASTP scores among protein sequences using also GET_HOMOLOGUES.

### Genes under positive selection and other analysis

We codon aligned the nucleotide sequences corresponding to each cluster found by GET_HOMOLOGUES and having at least four sequences by PRANK version 150803^60^. Alignments were then evaluated by GARD through HyPhy version 2.2.4^61^ to find potential recombination events. Finally, positive selection was evaluated with the PARRIS algorithm, also through HyPhy version 2.2.4.

Each genome was screened for antibiotic resistance genes by the RGI tool of the webserver CARD^62^. Homology searches were performed locally with BLASTP between all the CDSs annotated by RAST and the curated PATRIC_VF database^63^ which contained 1,570 sequences of CDSs known to be implicated in virulence (downloaded on July 8^th^ 2016) and also a database containing all integrase sequences available in the Protein database of the NCBI (1, 347, 621 sequences on July 8^th^, 2016).

### Host range

The host range of 12 phages was determined using a set of 65 isolates of *A. salmonicida* (see Supplementary Table S3). Cells stocks were thawed, streaked on TSA plates and grown for three days at 18 °C. Isolated colonies were inoculated in 3 ml TSB in sterile snap cap tubes and incubated overnight at 18 °C and agitated at 200 RPM. For each strain, 100 μl of overnight bacterial culture was added to 3 ml of soft agar and poured onto a TSA plate. Ten-fold serial dilutions of each phage (up to 10^−7^) were done in phage buffer and 5 μl of each dilution was spotted onto the inoculated TSA plates. The plates were incubated at 18 °C overnight and the sensitivity or insensitivity was recorded.

## Electronic supplementary material

Supplementary information file

Table S1

Table S2

Table S3
