# Supplementary material for: Characterization and diversity of phages infecting Aeromonas salmonicida subsp. salmonicida
Source: Sci Rep. 2017 Aug 1;7:7054. doi: 10.1038/s41598-017-07401-7 (PMC5539321; doi:10.1038/s41598-017-07401-7)
Supplement: Supplementary file 1 — Supplementary information file [file 41598_2017_7401_MOESM1_ESM.pdf]

Supplementary information file

Characterization and diversity of phages infecting *Aeromonas salmonicida* subsp. *salmonicida*

Antony T. Vincent[1,2,3], Valérie E. Paquet[1,2,3], Alex Bernatchez[1,2,3], Denise M. Tremblay[2,4,5], Sylvain Moineau[2,4,5] and Steve J. Charette[1,2,3,#]

1. Institut de Biologie Intégrative et des Systèmes (IBIS), Université Laval, Quebec City, QC, Canada, G1V 0A6
2. Département de biochimie, de microbiologie et de bio-informatique, Faculté des sciences et de génie, Université Laval, Quebec City, QC, Canada, G1V 0A6
3. Centre de recherche de l'Institut universitaire de cardiologie et de pneumologie de Québec (IUCPQ), Quebec City, QC, Canada, G1V 4G5
4. Groupe de Recherche en Écologie Buccale (GREB), Faculté de médecine dentaire, Université Laval, Quebec City, QC, Canada, G1V 0A6
5. Félix d'Hérelle Reference Center for Bacterial Viruses, Université Laval, Quebec City, QC, Canada, G1V 0A6

[#]To whom correspondence should be addressed: Institut de Biologie Intégrative et des Systèmes (IBIS), Pavillon Charles-Eugène-Marchand, 1030 avenue de la Médecine, Université Laval, Quebec City, QC, Canada, G1V 0A6

steve.charette@bcm.ulaval.ca; Telephone: 1-418-656-2131, ext. 6914; Fax: 1-418-656-7176

Figure S1 ……………………………………………………………………………Page 3

Figure S2 ……………………………………………………………………………Page 4

Figure S3 ……………………………………………………………………………Page 5

Figure S4 ……………………………………………………………………………Page 6

Figure S5 ……………………………………………………………………………Page 7

Figure S6 ……………………………………………………………………………Page 9

Figure S7 ……………………………………………………………………………Page 10

Figure S8 ……………………………………………………………………………Page 11

Figure S9 ……………………………………………………………………………Page 16

Figure S10 …………………………………………………………………………..Page 17

Figure S11 …………………………………………………………………………..Page 18

Figure S12 …………………………………………………………………………..Page 19

References …………………………………………………………………………..Page 20

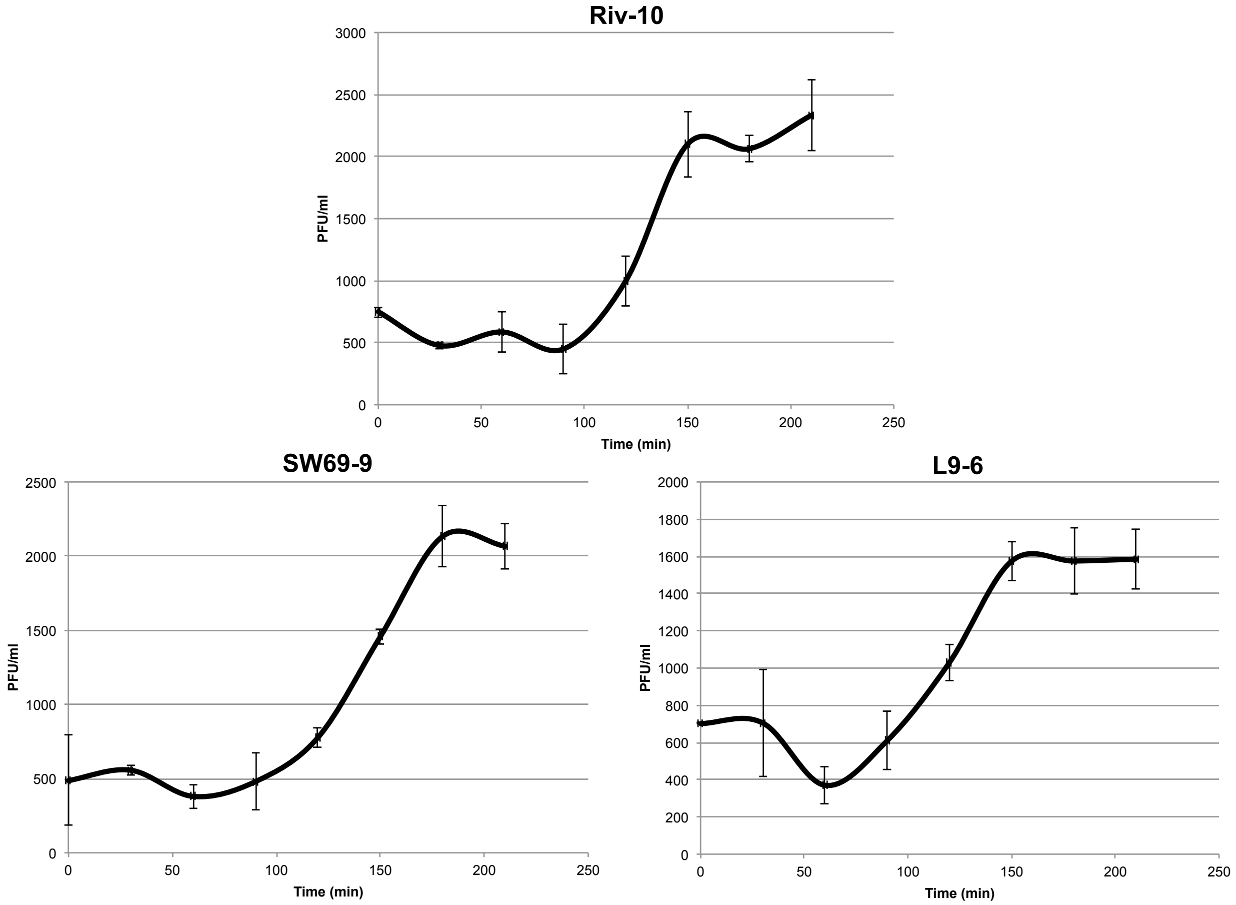

Figure S1. One-step growth curves for the newly isolated phages Riv-10, SW69-9 and L9-6.

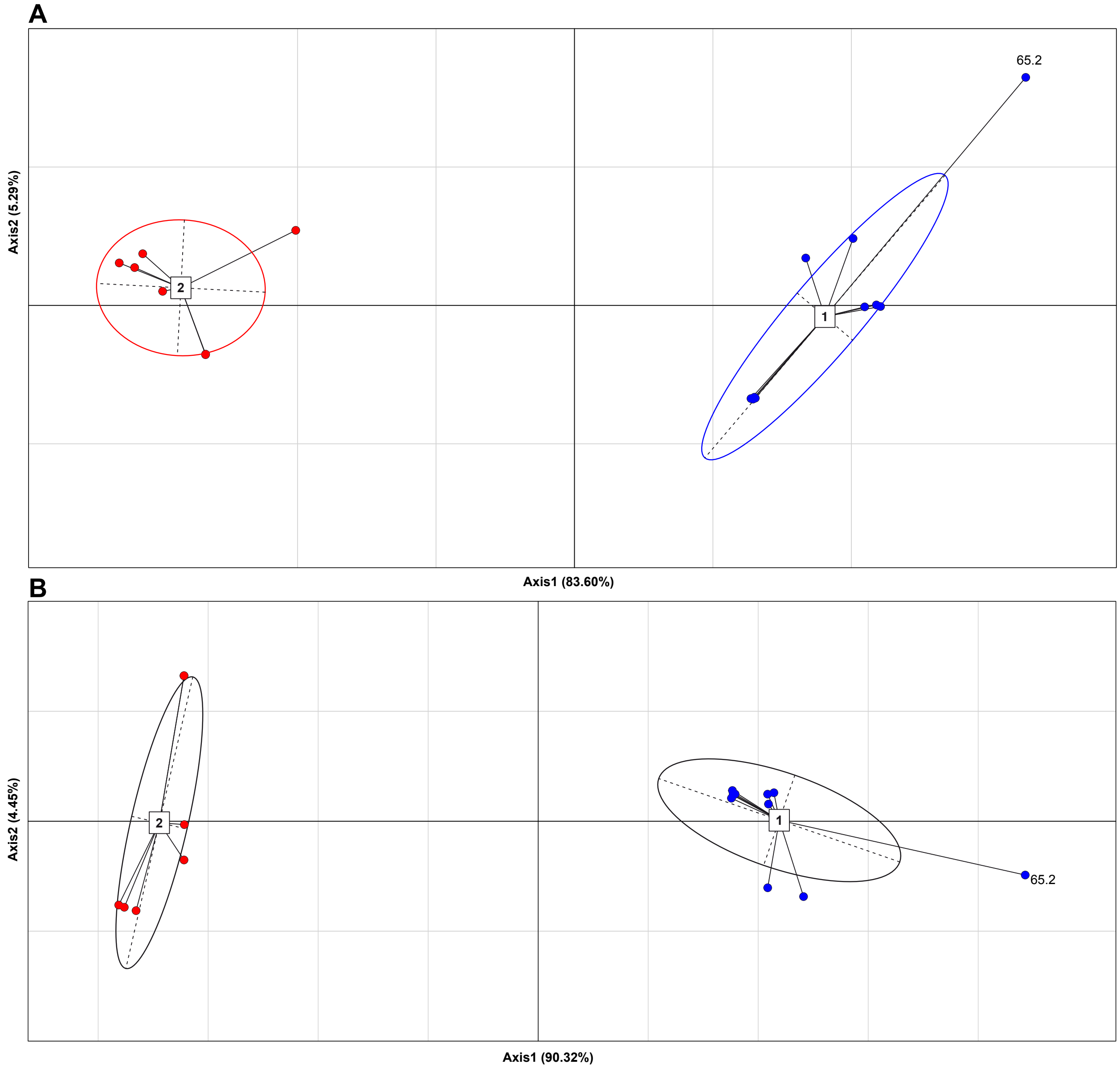

Figure S2. Principal component analysis (PCA) of the PCs 1 and 2 based on (A) the relative synonymous codon usage (RSCU) and (B) the amino acids composition. The genomes having low and high GC% are represented by the blue and red circles, respectively. The amino-acid composition of all CDSs of each phage genome was computed using pepstats of the EMBOSS package version 6.6.0.0[1] and the RSCU was determined by using DAMBE version 6.4.2[2]. PCA plots were produced by using the R packages ade4[3] and adegenet[4].

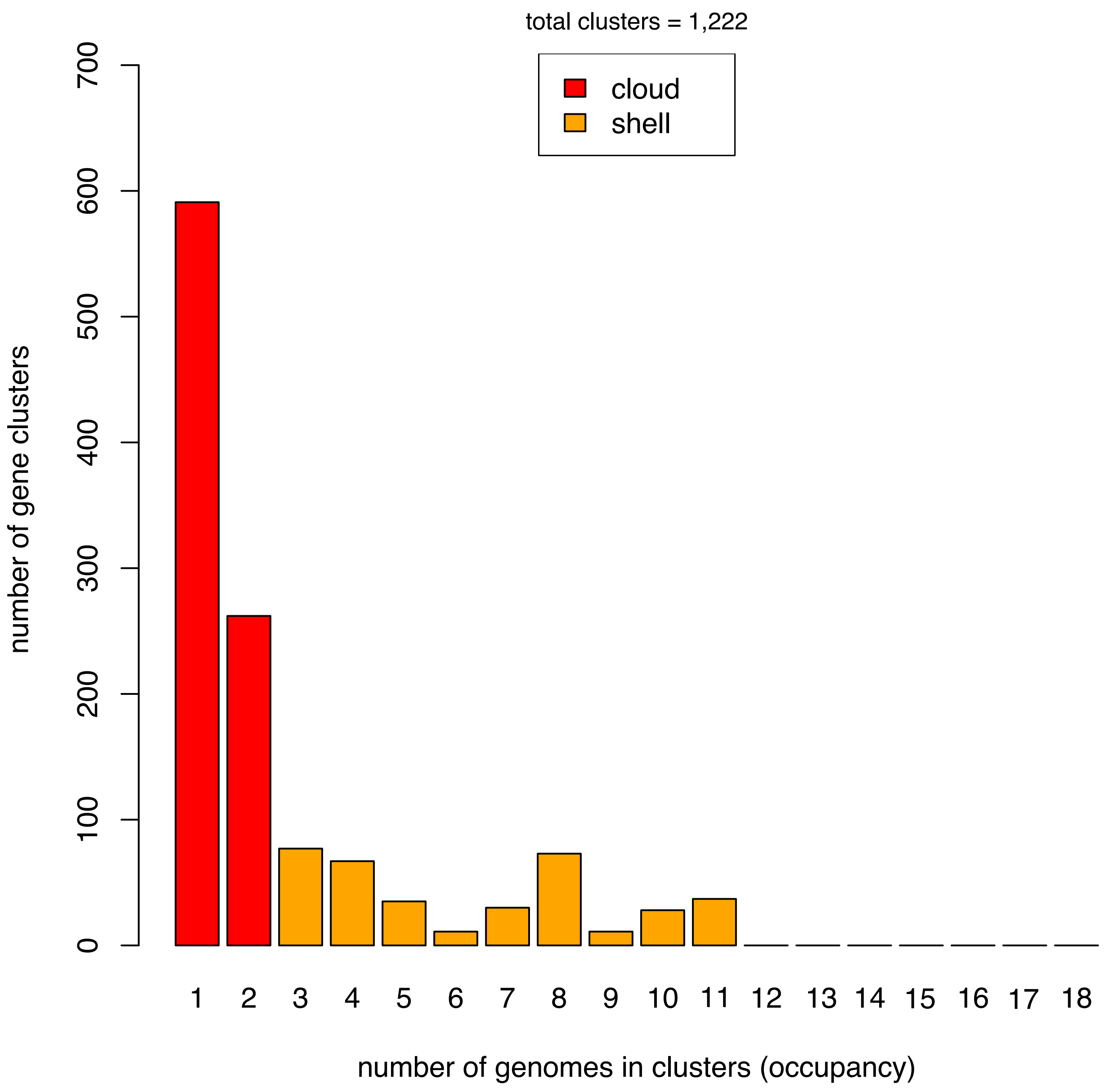

Figure S3. Pan-genome analysis for the 18 phage genomes used in the present study.

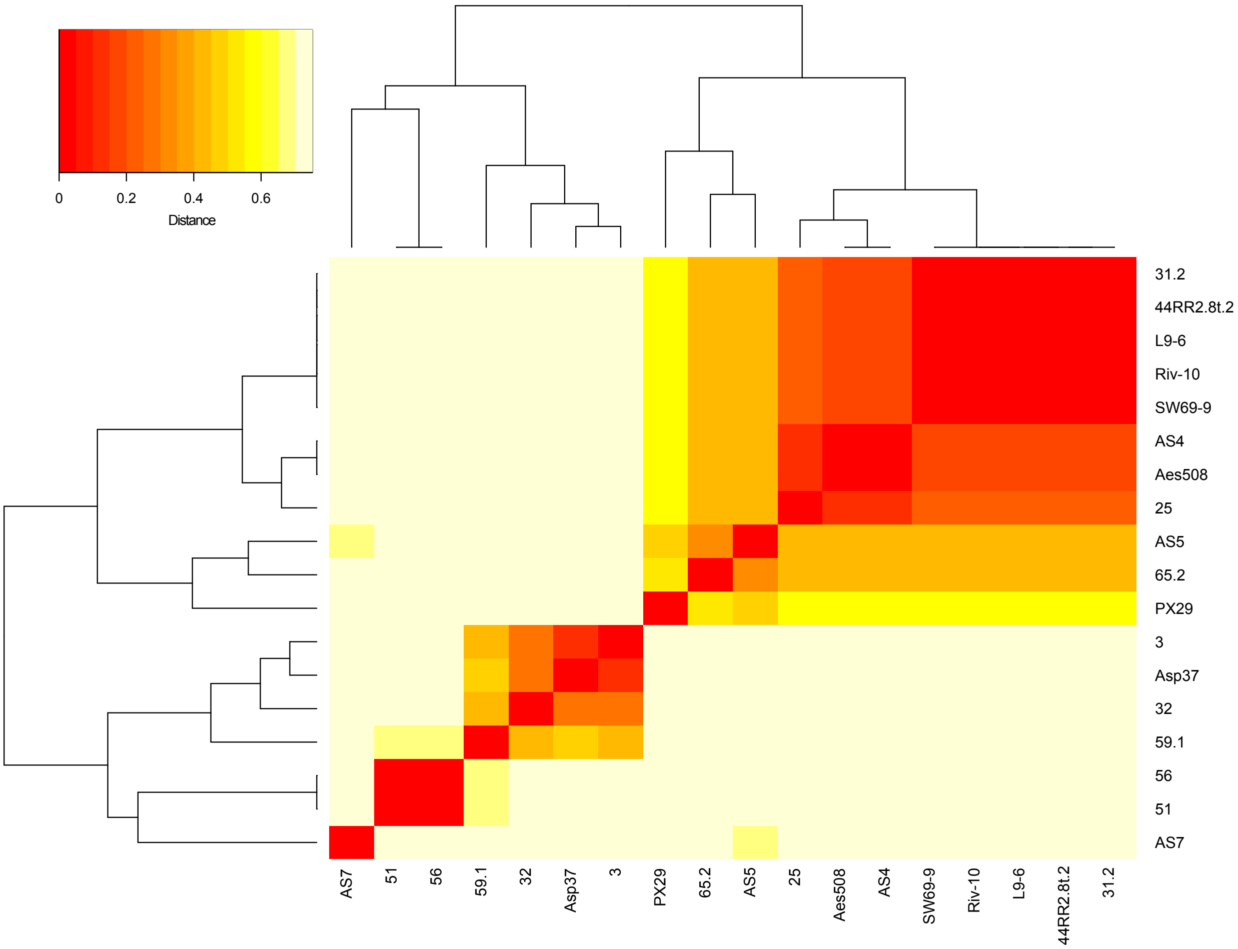

Figure S4. Clustering based on the identity distance of the terminase large subunit sequences.

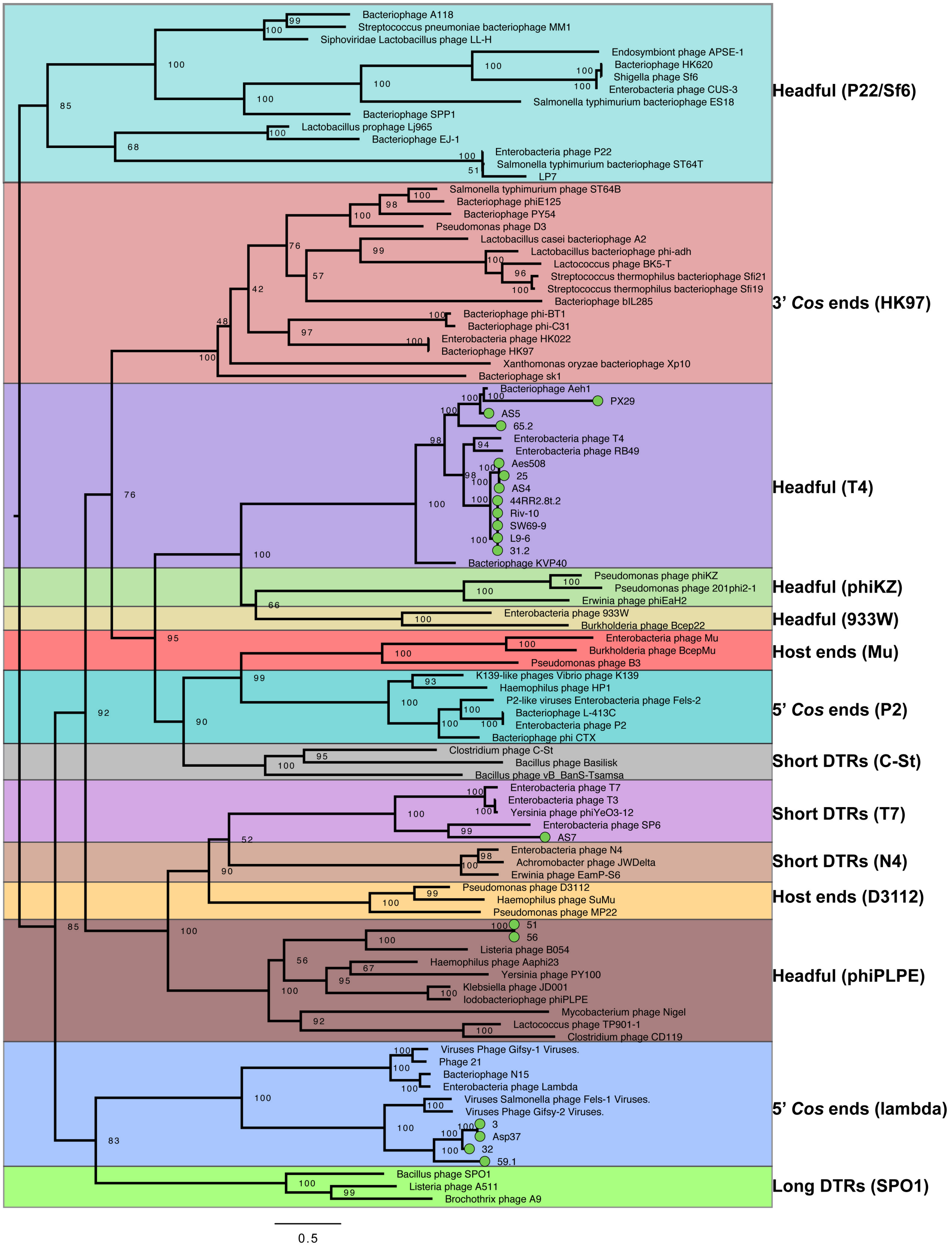

Figure S5. Molecular phylogeny based on the large terminase subunit sequences of 96 phages. Bootstrap values are indicated for all nodes. The cluster are coloured according to the DNA

packaging strategies. Green circles indicate phages of the present study. The dataset used was a derivative from two recently published studies[5,6]. Since the sequences were highly divergent, they were aligned by PROMALS3D[7], which uses structural information to guide the alignment. The resulting alignment was evaluated with IQ-TREE version 1.4.4[8] to find the best-fit model. Maximum-likelihood phylogenetic analysis was performed with IQ-TREE as well, using the model VT+F+R5 and 1000 ultrafast bootstrap (UFBoot)[9]. The tree was midpoint rooted by FigTree version 1.4.3 (http://tree.bio.ed.ac.uk/software/figtree/).

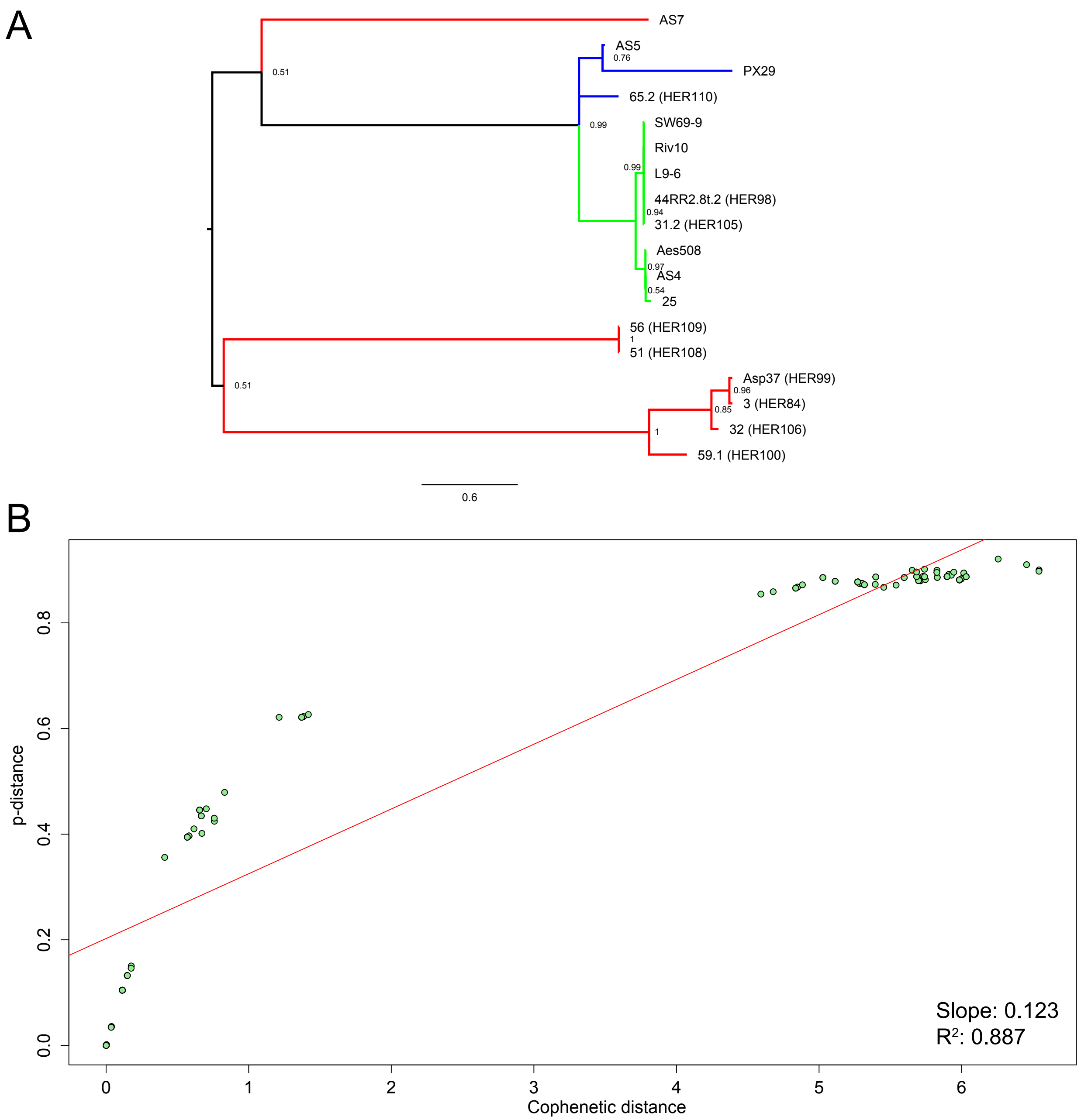

Figure S6. (A) Bayesian phylogenetic tree inferred with the site-heterogeneous model GTR+CAT. The phages having a small-, medium- and large-size genome are in red, green and blue, respectively. (B) Relation between the uncorrected p-distance and cophenetic distance. The molecular phylogeny was performed by Bayesian inference by running five independent chains under the model GTR+CAT for 10,000 cycles with PhyloBayes version 4.1[10]. A consensus topology was computed from the saved trees using BPCOMP included in the package PhyloBayes after a burn-in of 2 000 trees (20%). The largest discrepancy across all bipartitions (maxdiff) was 0.161375, meaning that the convergence between the chains was achieved. The resulting tree was used to test the level of saturation using an R script provided by another study[11].

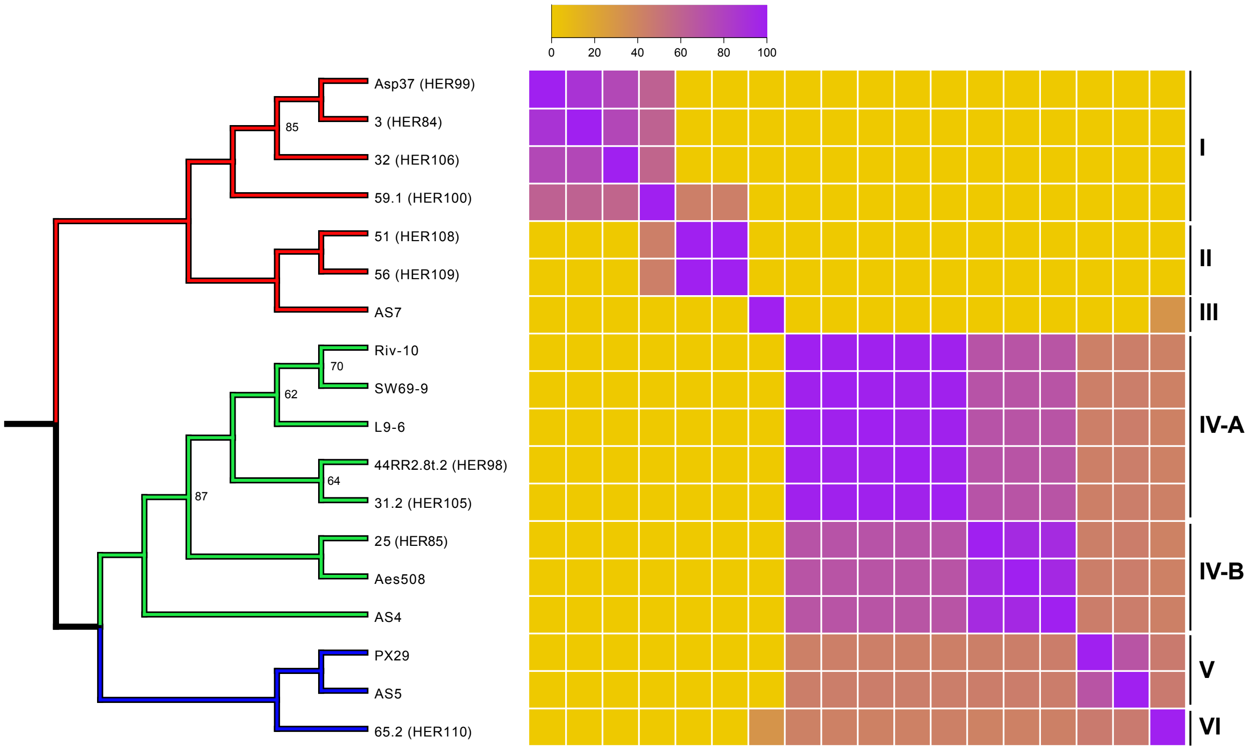

Figure S7. Hierarchical clustering of the phage genomes coupled with an identity matrix based on the translated protein-encoding genes.

Figure S8. Genomic comparisons between phages of group IV-A. Nucleotide identity shared with the reference genome (31.2 [HER105]) is shown in pink. Features (CDS and tRNAs) are displayed as arrows. Corresponding annotations of CDS and tRNAs are described on page 15. This analysis was produced with gVISTA online[12].

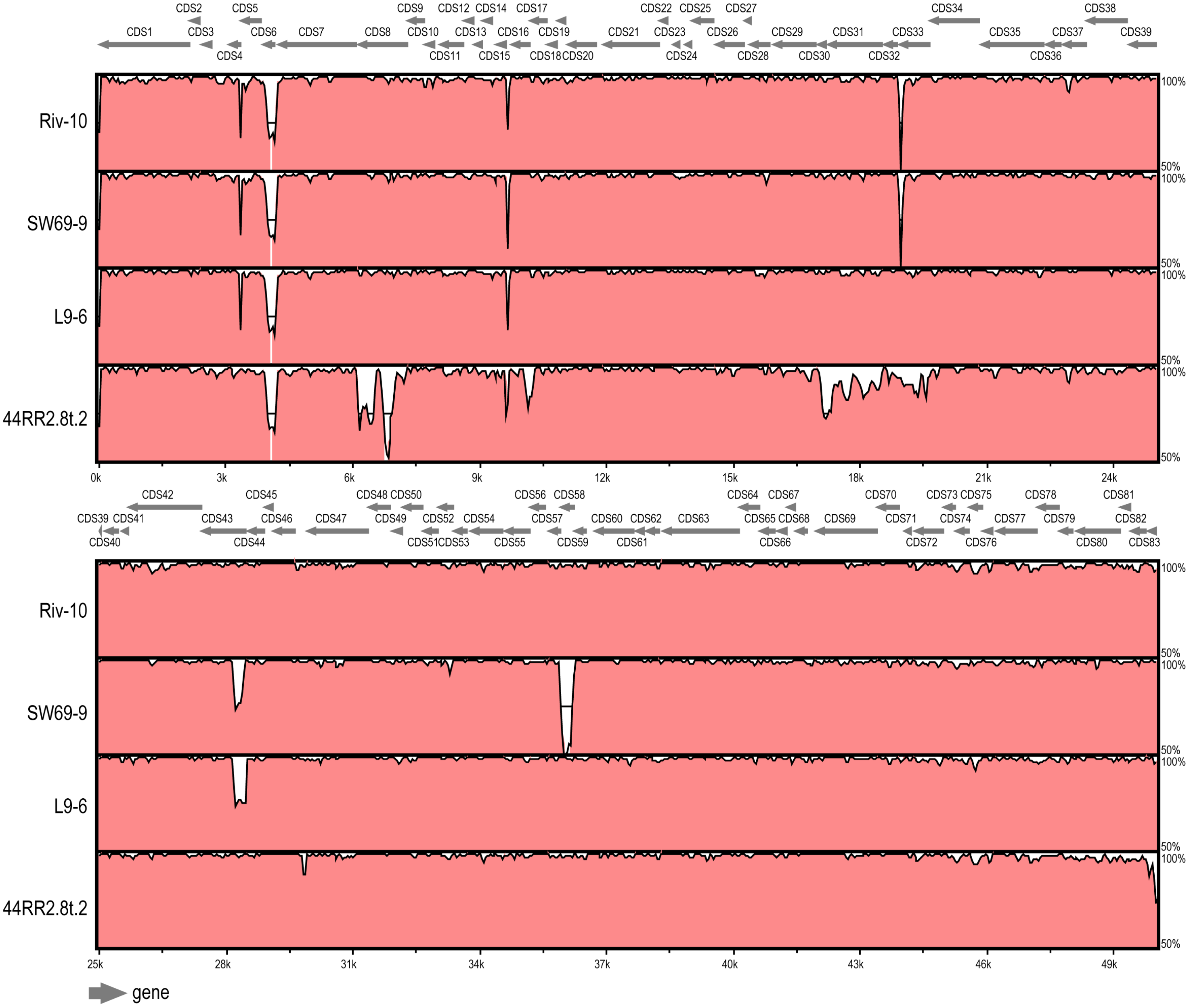

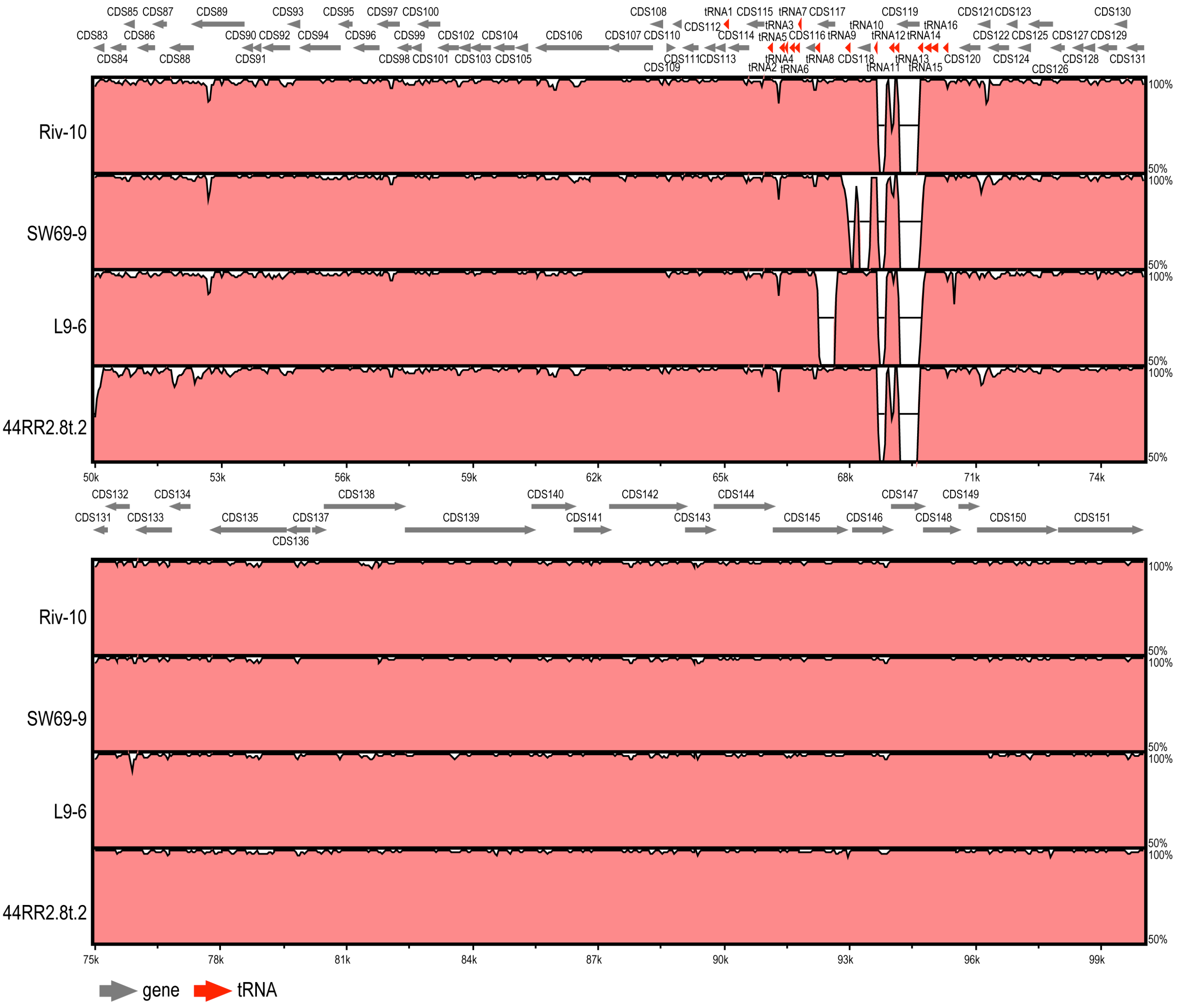

Riv-10
SW69-9
L9-6
44RR2.8t.2
100%
50%
50k
53k
56k
59k
62k
65k
68k
71k
74k
75k
78k
81k
84k
87k
90k
93k
96k
99k
gene
tRNA

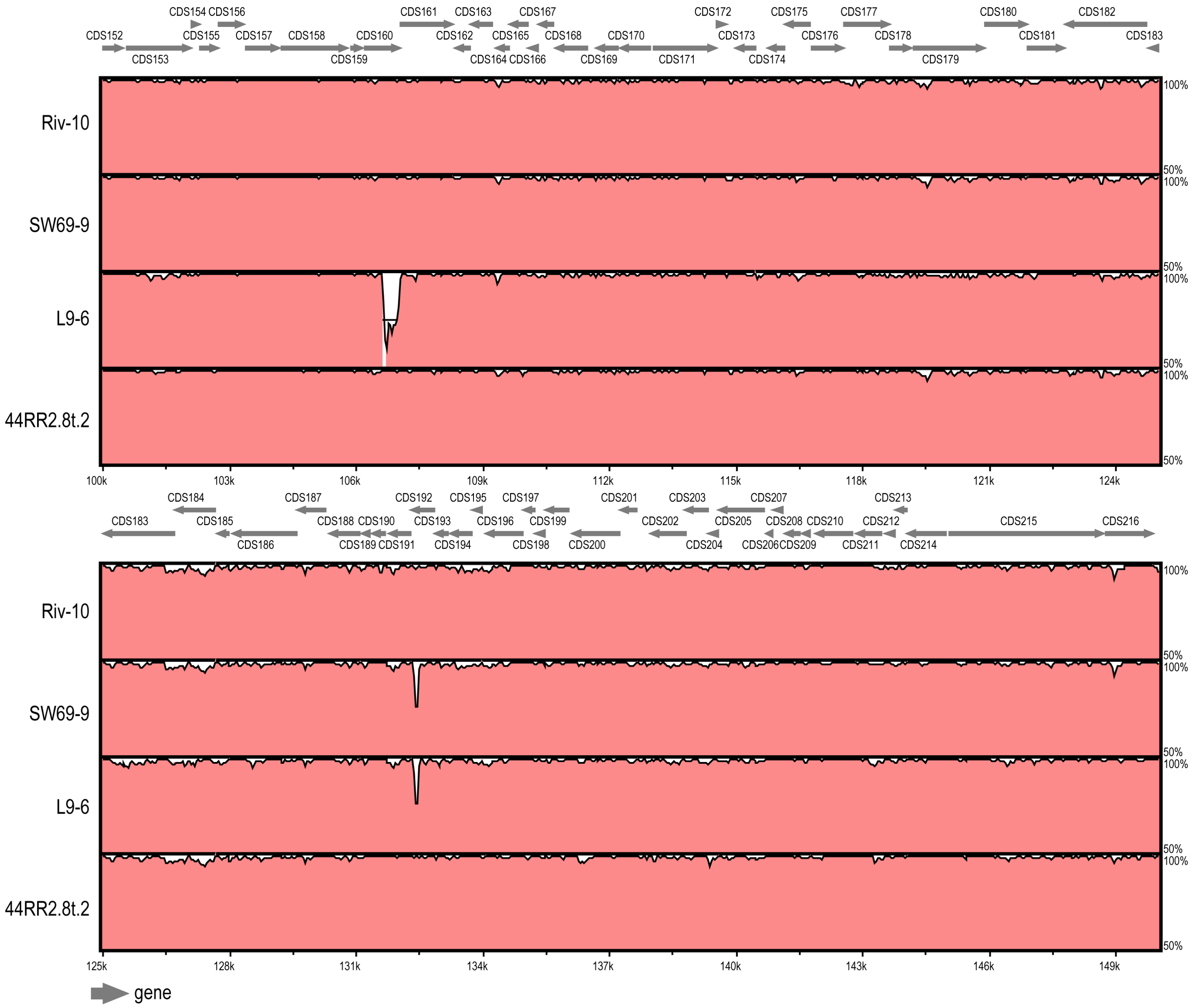

CDS152
CDS153
CDS154 CDS156
CDS155
CDS157
CDS158
CDS159
CDS160
CDS161
CDS162
CDS163
CDS164 CDS166
CDS165
CDS167
CDS168
CDS169
CDS170
CDS171
CDS172
CDS173
CDS174
CDS175
CDS176
CDS177
CDS178
CDS179
CDS180
CDS181
CDS182
CDS183
Riv-10
SW69-9
L9-6
44RR2.8t.2
100%
50%
100k
103k
106k
109k
112k
115k
118k
121k
124k
CDS183
CDS184
CDS185
CDS186
CDS187
CDS188 CDS190
CDS189 CDS191
CDS192
CDS193
CDS194
CDS195
CDS196
CDS197
CDS198
CDS199
CDS200
CDS201
CDS202
CDS203
CDS204
CDS205
CDS206CDS209
CDS207
CDS208
CDS210
CDS211
CDS212
CDS213
CDS214
CDS215
CDS216
Riv-10
SW69-9
L9-6
44RR2.8t.2
100%
50%
125k
128k
131k
134k
137k
140k
143k
146k
149k
gene

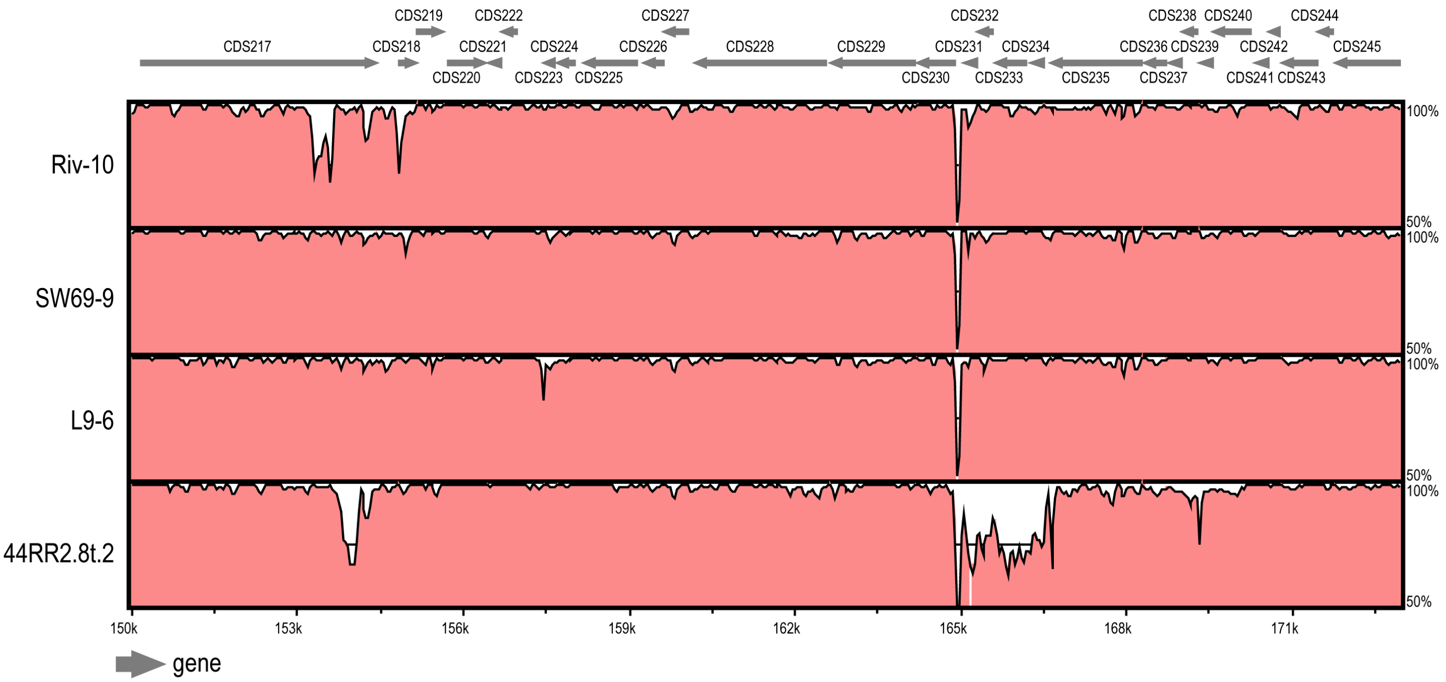
CDS217
CDS218
CDS219
CDS220
CDS221
CDS222
CDS223
CDS224
CDS225
CDS226
CDS227
CDS228
CDS229
CDS230
CDS231
CDS232
CDS233
CDS234
CDS235
CDS236
CDS237
CDS238
CDS239
CDS240
CDS241
CDS242
CDS243
CDS244
CDS245
Riv-10
SW69-9
L9-6
44RR2.8t.2
100%
50%
150k
153k
156k
159k
162k
165k
168k
171k
gene

Gene	Strand	Coordinates		Product
CDS1	-	1	2118	rIIA lysis inhibitor
CDS2	-	2125	2391	hypothetical protein
CDS3	-	2407	2673	hypothetical protein
CDS4	-	3076	3342	hypothetical protein
CDS5	-	3345	3806	hypothetical protein
CDS6	-	3878	4129	hypothetical protein
CDS7	-	4247	6070	DNA topoisomerase large subunit
CDS8	-	6124	7287	serine hydroxymethyltransferase
CDS9	-	7289	7705	hypothetical protein
CDS10	-	7707	7943	hypothetical protein
CDS11	-	8072	8617	hypothetical protein
CDS12	-	8614	8853	hypothetical protein
CDS13	-	8850	9068	hypothetical protein
CDS14	-	9065	9307	hypothetical protein
CDS15	-	9391	9606	modifier of suppressor tRNAs
CDS16	-	9728	10204	hypothetical protein
CDS17	-	10189	10578	hypothetical protein
CDS18	-	10575	10820	hypothetical protein
CDS19	-	10817	11017	hypothetical protein
CDS20	-	11074	11739	exonuclease A
CDS21	-	11911	13230	DNA helicase
CDS22	-	13233	13457	hypothetical protein
CDS23	-	13561	13740	hypothetical protein
CDS24	-	13855	13998	hypothetical protein
CDS25	-	13998	14516	dCTP pyrophosphatase
CDS26	-	14576	15241	hypothetical protein
CDS27	-	15238	15408	hypothetical protein
CDS28	-	15398	15856	endonuclease
CDS29	-	15952	16956	DNA primase
CDS30	-	16997	17182	hypothetical protein
CDS31	-	17217	18518	DNA primase/helicase
CDS32	-	18554	18895	capsid and scaffold protein
CDS33	-	18932	19627	deoxycytidylate 5-hydroxymethyltransferase
CDS34	-	19637	20812	DNA polymerase
CDS35	-	20862	22358	DNA polymerase
CDS36	-	22402	22758	RegA
CDS37	-	22768	23346	DNA polymerase clamp loader subunit
CDS38	-	23343	24302	replication factor C small subunit
CDS39	-	24374	25045	sliding clamp DNA polymerase accessory protein
CDS40	-	25103	25456	RNA polymerase
CDS41	-	25515	25703	hypothetical protein
CDS42	-	25693	27405	recombination-related endonuclease
CDS43	-	27402	28469	recombination-related endonuclease
CDS44	-	28520	28912	hypothetical protein
CDS45	-	28902	29126	hypothetical protein
CDS46	-	29104	29622	RNA polymerase sigma factor for late transcription
CDS47	-	29923	31362	hypothetical protein
CDS48	-	31359	31865	hypothetical protein
CDS49	-	31922	32176	hypothetical protein
CDS50	-	32176	32643	hypothetical protein
CDS51	-	32643	32999	hypothetical protein
CDS52	-	33000	33362	hypothetical protein
CDS53	-	33359	33697	hypothetical protein
CDS54	-	33765	34556	hypothetical protein
CDS55	-	34598	35185	hypothetical protein
CDS56	-	35196	35546	hypothetical protein
CDS57	-	35646	35921	glutaredoxin
CDS58	-	35918	36226	hypothetical protein
CDS59	-	36223	36534	thioredoxin
CDS60	-	36728	37630	hypothetical protein
CDS61	-	37675	37893	hypothetical protein
CDS62	-	37906	38226	hypothetical protein
CDS63	-	38306	40132	ribonucleotide reductase of class III (anaerobic) large subunit
CDS64	-	40129	40602	endonuclease
CDS65	-	40632	40979	hypothetical protein
CDS66	-	41005	41268	hypothetical protein
CDS67	-	41271	41477	hypothetical protein
CDS68	-	41462	41737	hypothetical protein
CDS69	-	41952	43406	nicotinamide phosphoribosyltransferase
CDS70	-	43409	43912	hypothetical protein
CDS71	-	44056	44202	hypothetical protein
CDS72	-	44276	44977	hypothetical protein
CDS73	-	44956	45252	hypothetical protein
CDS74	-	45252	45575	hypothetical protein
CDS75	-	45572	45883	hypothetical protein
CDS76	-	45880	46125	hypothetical protein
CDS77	-	46200	47174	thioredoxin
CDS78	-	47184	47690	hypothetical protein
CDS79	-	47687	48031	hypothetical protein
CDS80	-	48104	49162	NadM family nicotinamide-nucleotide adenylyltransferase
CDS81	-	49164	49409	hypothetical protein
CDS82	-	49406	49774	hypothetical protein
CDS83	-	49816	50199	hypothetical protein
CDS84	-	50400	50717	hypothetical protein
CDS85	-	50719	50919	hypothetical protein
CDS86	-	51033	51413	hypothetical protein
CDS87	-	51394	51678	lysis inhibition regulator

Gene	Strand	Coordinates		Product
CDS88	-	51831	52340	hypothetical protein
CDS89	-	52340	53530	hypothetical protein
CDS90	-	53530	53760	hypothetical protein
CDS91	-	53770	53958	hypothetical protein
CDS92	-	54033	54641	lipoprotein
CDS93	-	54646	54864	hypothetical protein
CDS94	-	54918	55841	hypothetical protein
CDS95	-	55857	56120	hypothetical protein
CDS96	-	56193	56768	thymidine kinase
CDS97	-	56768	57241	hypothetical protein
CDS98	-	57231	57542	hypothetical protein
CDS99	-	57560	57757	hypothetical protein
CDS100	-	57744	58232	hypothetical protein
CDS101	-	58229	58669	hypothetical protein
CDS102	-	58693	58920	hypothetical protein
CDS103	-	58985	59404	hypothetical protein
CDS104	-	59533	59982	endoribonuclease
CDS105	-	60081	60308	hypothetical protein
CDS106	-	60528	62240	ribonucleotide reductase of class Ia (aerobic) alpha subunit
CDS107	-	62292	63290	ribonucleotide reductase of class Ia (aerobic) beta subunit
CDS108	-	63300	63533	hypothetical protein
CDS109	+	63660	63818	hypothetical protein
CDS110	-	63807	63980	hypothetical protein
CDS111	-	64066	64359	hypothetical protein
CDS112	-	64586	64789	hypothetical protein
CDS113	-	64800	65009	hypothetical protein
tRNA1	-	65030	65103	tRNA-Arg
CDS114	-	65121	65570	hypothetical protein
CDS115	-	65587	65955	pyruvate formate-lyase
tRNA2	-	66075	66146	tRNA-Met
tRNA3	-	66356	66429	tRNA-Met
tRNA4	-	66452	66524	tRNA-Asp
tRNA5	-	66601	66685	tRNA-Ser
tRNA6	-	66696	66768	tRNA-Ile
tRNA7	-	66774	66846	tRNA-Trp
CDS116	-	66994	67170	hypothetical protein
tRNA8	-	67198	67270	tRNA-Thr
CDS117	-	67272	67631	hypothetical protein
tRNA9	-	67913	67986	tRNA-Pro
CDS118	-	68231	68470	hypothetical protein
tRNA10	-	68583	68655	tRNA-His
tRNA11	-	68950	69022	tRNA-Lys
tRNA12	-	69083	69162	tRNA-Tyr
CDS119	-	69164	69631	hypothetical protein
tRNA13	-	69661	69743	tRNA-Asn
tRNA14	-	69825	69909	tRNA-Phe
tRNA15	-	69983	70073	tRNA-Ser
tRNA16	-	70267	70347	tRNA-Leu
CDS120	-	70670	71110	hypothetical protein
CDS121	-	71111	71356	hypothetical protein
CDS122	-	71353	71772	hypothetical protein
CDS123	-	71785	71997	hypothetical protein
CDS124	-	72062	72322	hypothetical protein
CDS125	-	72322	72819	hypothetical protein
CDS126	-	72826	73092	hypothetical protein
CDS127	-	73343	73564	hypothetical protein
CDS128	-	73594	73848	hypothetical protein
CDS129	-	73944	74372	hypothetical protein
CDS130	-	74372	74608	tail fibers chaperone
CDS131	-	74622	75296	deoxynucleotide monophosphate kinase
CDS132	-	75298	75825	tail completion protein
CDS133	-	76011	76817	DNA end protector during packaging protein
CDS134	-	76817	77269	head completion protein
CDS135	-	77767	79569	baseplate hub protein
CDS136	-	79575	80114	baseplate wedge subunit
CDS137	+	80184	80477	phospholipase
CDS138	+	80474	82357	baseplate wedge subunit
CDS139	+	82389	85448	baseplate wedge subunit
CDS140	+	85441	86427	baseplate wedge subunit
CDS141	+	86437	87294	baseplate wedge tail fiber connector
CDS142	+	87294	89108	baseplate wedge subunit and tail pin
CDS143	+	89108	89770	baseplate wedge subunit and tail pin
CDS144	+	89770	91170	tail fibers
CDS145	+	91167	92930	neck whiskers
CDS146	+	93091	94014	head completion, neck hetero-dimeric protein
CDS147	+	94021	94779	head completion, neck hetero-dimeric protein
CDS148	+	94782	95600	proximal tail sheath stabilization protein
CDS149	+	95604	96068	terminase small subunit
CDS150	+	96068	97909	terminase large subunit
CDS151	+	97969	99960	tail sheath monomer
CDS152	+	100014	100502	tail fibers
CDS153	+	100546	102096	portal vertex of the head
CDS154	+	102109	102306	prohead core protein
CDS155	+	102307	102729	capsid and scaffold protein
CDS156	+	102729	103361	prohead assembly (scaffolding) protein
CDS157	+	103371	104195	prohead assembly (scaffolding) protein
CDS158	+	104217	105806	major capsid protein

Gene	Strand	Coordinates		Product
CDS159	+	105885	106172	hypothetical protein
CDS160	+	106183	107049	hypothetical protein
CDS161	+	107061	108293	capsid vertex protein
CDS162	-	108342	108710	hypothetical protein
CDS163	-	108707	109231	postulated decoy of host sigma70 or sigmaS
CDS164	-	109299	109607	hypothetical protein
CDS165	-	109607	110074	guanosine-3',5'-bis(Diphosphate) 3'-pyrophosphohydrolase
CDS166	-	110074	110313	hypothetical protein
CDS167	-	110319	110657	hypothetical protein
CDS168	-	110717	111478	hypothetical protein
CDS169	-	111665	112207	capsid and scaffold protein
CDS170	-	112247	112981	protein inh
CDS171	+	113036	114517	DNA helicase
CDS172	+	114527	114757	DNA helicase
CDS173	-	114971	115441	hypothetical protein
CDS174	-	115749	116132	baseplate wedge subunit
CDS175	-	116129	116743	baseplate hub assembly chaperone
CDS176	+	116790	117536	baseplate protein
CDS177	+	117536	118651	baseplate hub protein
CDS178	+	118648	119175	baseplate hub protein
CDS179	+	119185	120885	baseplate hub protein
CDS180	+	120882	121910	baseplate tail tube cap
CDS181	+	121912	122769	baseplate tail tube initiator
CDS182	-	122797	124725	RNA polymerase-ADP-ribosyltransferase
CDS183	-	124755	126674	RNA polymerase-ADP-ribosyltransferase
CDS184	-	126706	127665	hypothetical protein
CDS185	-	127708	127974	hypothetical protein
CDS186	-	128077	129582	DNA ligase
CDS187	-	129579	130283	hypothetical protein
CDS188	-	130352	131062	hypothetical protein
CDS189	-	131134	131325	hypothetical protein
CDS190	-	131356	131694	head assembly chaperone
CDS191	-	131756	132280	tail fibers
CDS192	-	132290	132850	hypothetical protein
CDS193	-	132847	133161	hypothetical protein
CDS194	-	133219	133737	dCMP deaminase
CDS195	-	133737	133967	hypothetical protein
CDS196	-	134077	134964	3'-phosphatase, 5'-polynucleotide kinase
CDS197	-	134961	135209	hypothetical protein
CDS198	-	135206	135469	hypothetical protein
CDS199	-	135480	136016	transcription terminator
CDS200	-	136094	137245	RNA ligase
CDS201	-	137242	137658	endonuclease
CDS202	-	137951	138790	thymidylate synthase
CDS203	-	138787	139341	dihydrofolate reductase
CDS204	-	139338	139571	hypothetical protein
CDS205	-	139573	140673	hypothetical protein
CDS206	-	140684	140842	hypothetical protein
CDS207	-	140845	141084	hypothetical protein
CDS208	-	141128	141517	hypothetical protein
CDS209	-	141528	141761	hypothetical protein
CDS210	-	141877	142764	single stranded DNA-binding protein
CDS211	-	142835	143434	DNA helicase loader
CDS212	-	143503	143760	transcriptional regulator
CDS213	-	143760	144047	double-stranded DNA binding protein
CDS214	-	144057	144980	ribonuclease H
CDS215	+	145055	148723	long tail fiber proximal subunit
CDS216	+	148733	149866	tail fiber protein
CDS217	+	150155	154414	tail fiber protein
CDS218	+	154820	155143	hypothetical protein
CDS219	+	155153	155647	baseplate hub protein
CDS220	+	155719	156384	holin
CDS221	-	156428	156688	hypothetical protein
CDS222	-	156698	156970	anti-sigma factor
CDS223	-	157446	157637	hypothetical protein
CDS224	-	157689	158021	hypothetical protein
CDS225	-	158163	159140	thioredoxin
CDS226	-	159240	159629	hypothetical protein
CDS227	-	159639	160076	hypothetical protein
CDS228	-	160177	162564	hypothetical protein
CDS229	-	162634	164157	putative sodium-dependent galactose transporter
CDS230	-	164224	164877	transcriptional regulator of middle promoters
CDS231	-	165060	165293	hypothetical protein
CDS232	-	165290	165598	hypothetical protein
CDS233	-	165608	166186	hypothetical protein
CDS234	-	166251	166493	hypothetical protein
CDS235	-	166628	168292	DNA topoisomerase
CDS236	-	168302	168739	nucleoid disruption protein
CDS237	-	168753	169013	hypothetical protein
CDS238	-	169013	169270	hypothetical protein
CDS239	-	169328	169579	hypothetical protein
CDS240	-	169579	170265	hypothetical protein
CDS241	-	170332	170556	hypothetical protein
CDS242	-	170553	170759	hypothetical protein
CDS243	-	170812	171465	hypothetical protein
CDS244	-	171462	171752	hypothetical protein
CDS245	-	171798	172931	lysis inhibitor

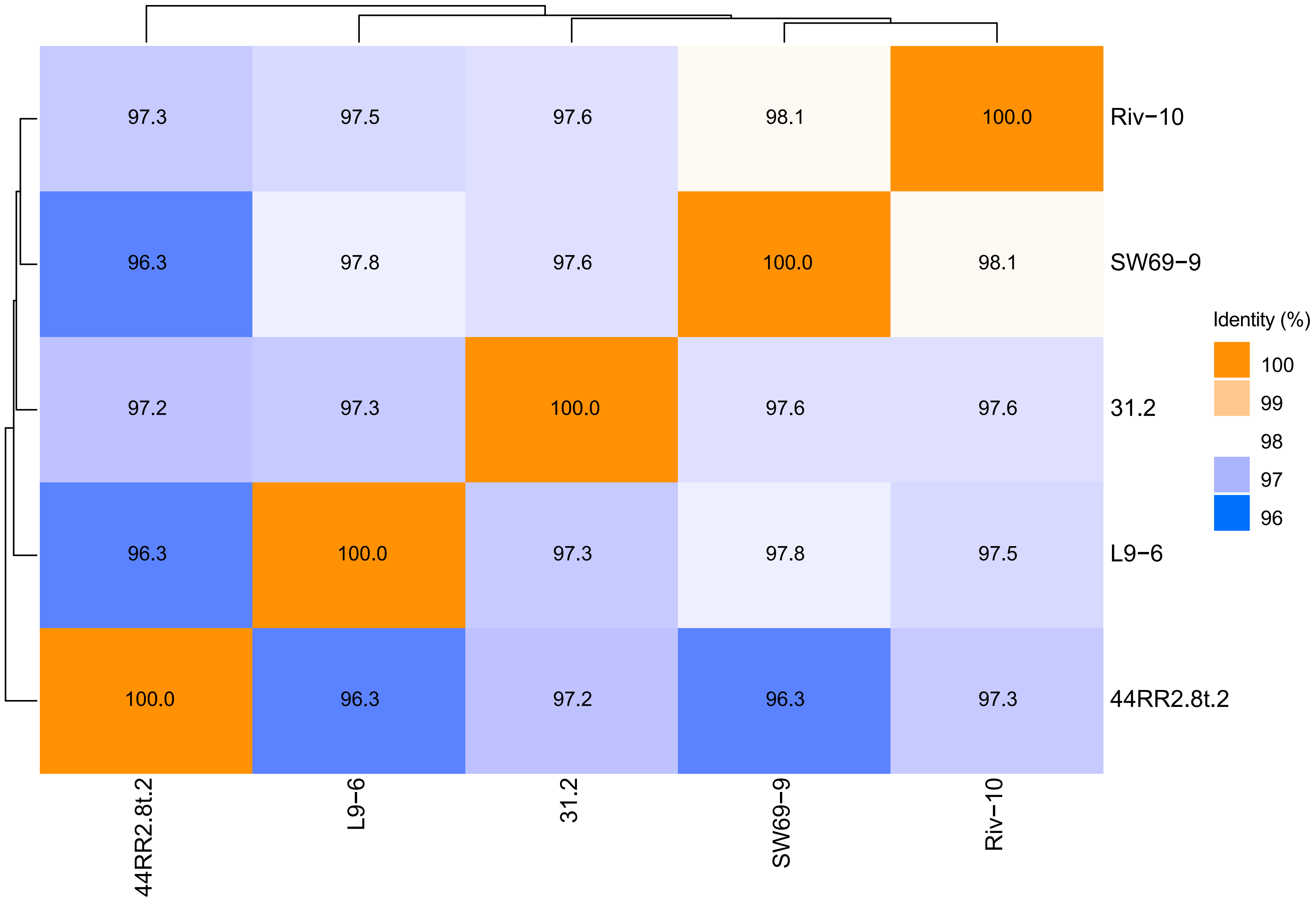

Figure S9. Whole genome comparisons between the newly isolated phages Riv-10, SW69-9, L9-6 and the two other phage genomes from the group IV-A. Global alignments were done using Stretcher from EMBOSS 6.6.0.0 [1].

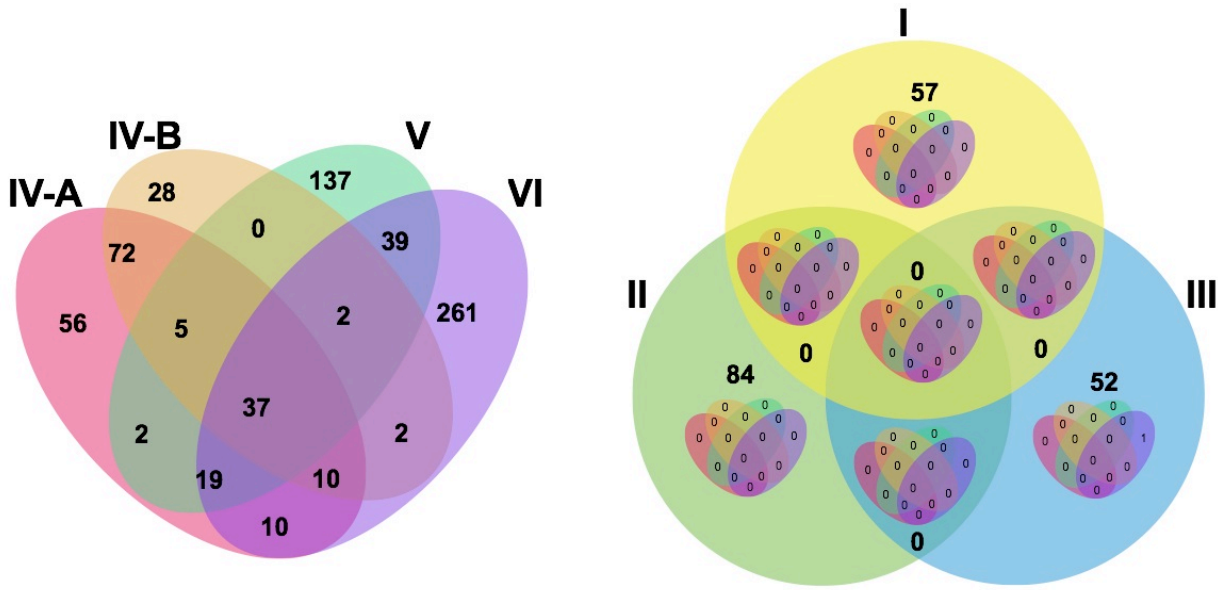

Figure S10. Numbers of shared core-clusters between the groups of studied phages. Venn diagrams were produced using VennPainter version 1.2.0[13]

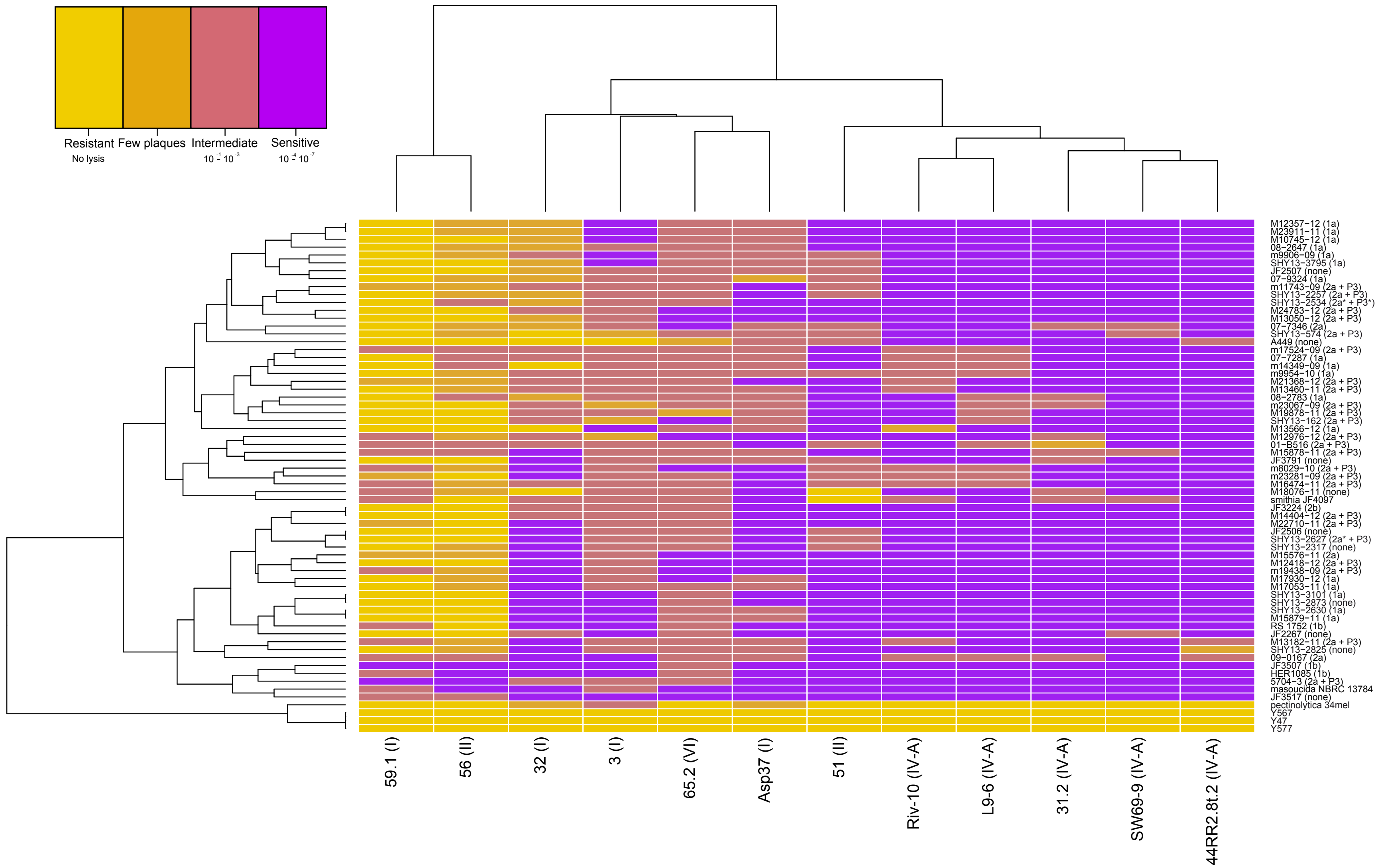

Figure S11. Clustering and heatmap based on a panel of 65 *A. salmonicida* isolates challenged with the 12 phages from this study. The genomic cluster of each phage is indicated in parentheses. The variants of *AsaGEI* (*1a*, *1b*, *2a* and *2b*) and the presence of the prophage 3 are indicated between parentheses for each isolates of the *salmonicida* subspecies. High and low lytic activities are represented in purple and yellow, respectively.

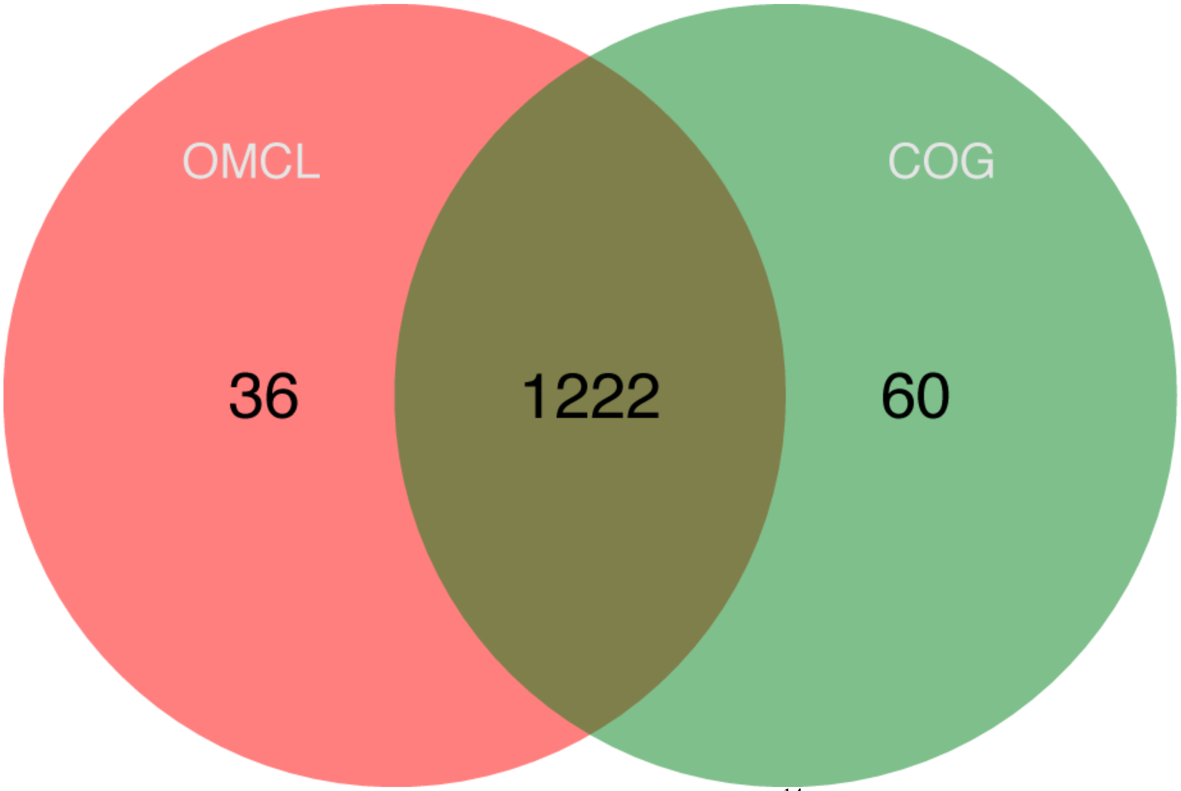

Figure S12. Gene clusters found by GET_HOMOLOGUES [14] depending of the algorithm used (OMCL[15] or COG[16]).

References

1. Rice, P., Longden, I. & Bleasby, A. EMBOSS: The European Molecular Biology Open Software Suite. *Trends Genet.* **16,** 276–277 (2000).
2. Xia, X. DAMBE5: A comprehensive software package for data analysis in molecular biology and evolution. *Mol. Biol. Evol.* **30,** 1720–1728 (2013).
3. Dray, S. & Dufour, A. B. The ade4 Package: Implementing the Duality Diagram for Ecologists. *J. Stat. Softw.* **22,** 1 – 20 (2007).
4. Jombart, T. Adegenet: A R package for the multivariate analysis of genetic markers. *Bioinformatics* **24,** 1403–1405 (2008).
5. Cheepudom, J., Lee, C. C., Cai, B. & Meng, M. Isolation, characterization, and complete genome analysis of P1312, a thermostable bacteriophage that infects *Thermobifida fusca*. *Front. Microbiol.* **6,** (2015).
6. Merrill, B. D., Ward, A. T., Grose, J. H. & Hope, S. Software-based analysis of bacteriophage genomes, physical ends, and packaging strategies. *BMC Genomics* **17,** 679 (2016).
7. Pei, J., Kim, B. H. & Grishin, N. V. PROMALS3D: A tool for multiple protein sequence and structure alignments. *Nucleic Acids Res.* **36,** 2295–2300 (2008).
8. Nguyen, L. T., Schmidt, H. A., Von Haeseler, A. & Minh, B. Q. IQ-TREE: A fast and effective stochastic algorithm for estimating maximum-likelihood phylogenies. *Mol. Biol. Evol.* **32,** 268–274 (2015).
9. Minh, B. Q., Nguyen, M. A. T. & Von Haeseler, A. Ultrafast approximation for phylogenetic bootstrap. *Mol. Biol. Evol.* **30,** 1188–1195 (2013).
10. Lartillot, N., Lepage, T. & Blanquart, S. PhyloBayes 3: a Bayesian software package for phylogenetic reconstruction and molecular dating. *Bioinformatics* **25,** 2286–8 (2009).
11. Borowiec, M. L., Lee, E. K., Chiu, J. C. & Plachetzki, D. C. Extracting phylogenetic signal and accounting for bias in whole-genome data sets supports the Ctenophora as sister to remaining Metazoa. *BMC Genomics* **16,** 987 (2015).
12. Couronne, O. *et al.* Strategies and tools for whole-genome alignments. *Genome Res.* **13,** 73–80 (2003).
13. Lin, G. *et al.* VennPainter: A Tool for the Comparison and Identification of Candidate Genes Based on Venn Diagrams. *PLoS One* **11,** e0154315 (2016).
14. Contreras-Moreira, B. & Vinuesa, P. GET_HOMOLOGUES, a versatile software package for scalable and robust microbial pangenome analysis. *Appl. Environ. Microbiol.* **79,** 7696–7701 (2013).
15. Li, L., Stoeckert, C. J. & Roos, D. S. OrthoMCL: Identification of ortholog groups for eukaryotic genomes. *Genome Res.* **13,** 2178–2189 (2003).
16. Kristensen, D. M. *et al.* A low-polynomial algorithm for assembling clusters of orthologous groups from intergenomic symmetric best matches. *Bioinformatics* **26,** 1481–1487 (2010).
